# LncRNA *IRAR* regulates chemokines production in tubular epithelial cells thus promoting kidney ischemia-reperfusion injury

**DOI:** 10.1038/s41419-022-05018-x

**Published:** 2022-06-22

**Authors:** Ping Jia, Sujuan Xu, Ting Ren, Tianyi Pan, Xiaoyan Wang, Yunlu Zhang, Zhouping Zou, Man Guo, Qi Zeng, Bo Shen, Xiaoqiang Ding

**Affiliations:** 1 Division of Nephrology, Zhongshan Hospital, Fudan University, Shanghai, China; 2 Shanghai Medical Center of Kidney, Shanghai, China; 3 Kidney and Dialysis Institute of Shanghai, Shanghai, China; 4 Kidney and Blood Purification Laboratory of Shanghai, Shanghai, China; 5 Hemodialysis quality control center of Shanghai, Shanghai, China

**Keywords:** Acute kidney injury, Long non-coding RNAs

## Abstract

Increasing evidence demonstrates that long noncoding RNAs (lncRNAs) play an important role in several pathogenic processes of the kidney. However, functions of lncRNAs in ischemic acute kidney injury (AKI) remain undefined. In this study, global lncRNA profiling indicated that many lncRNA transcripts were deregulated in kidney after ischemia reperfusion (IR). Among them, we identified *IRAR* (ischemia-reperfusion injury associated RNA) as a potential lncRNA candidate, which was mostly expressed by the tubular epithelial cells (TECs) after IR, involved in the development of AKI. GapmeR-mediated silencing and viral-based overexpression of *IRAR* were carried out to assess its function and contribution to IR-induced AKI. The results revealed that in vivo silencing of *IRAR* significantly reduced IR-induced proinflammatory cells infiltration and AKI. *IRAR* overexpression induced chemokine CCL2, CXCL1 and CXCL2 expression both in mRNA and protein levels in TECs, while, silencing of *IRAR* resulted in downregulation of these chemokines. RNA immunoprecipitation and RNA pulldown assay validated the association between *IRAR* and CCL2, CXCL1/2. Further examination revealed that specific ablation of CCL2 in TECs reduced macrophages infiltration and proinflammatory cytokine production, attenuated renal dysfunction in IR mice. Inhibition of CXC chemokine receptor 2 (receptor of CXCL1/2) reduced neutrofils infiltration, but had no overt effect on kidney function. To explore the mechanism of *IRAR* upregulation in kidney during IR, we analyzed promoter region of *IRAR* and predicted a potential binding site for transcription factor C/EBP β on *IRAR* promoter. Silencing of C/EBP β reduced *IRAR* expression in TECs. A dual-luciferase reporter assay and chromatin immunoprecipitation (ChIP) confirmed that *IRAR* was a transcriptional target of the C/EBP β. Altogether, our findings identify *IRAR* as a new player in the development of ischemic AKI through regulating chemokine production and immune cells infiltration, suggesting that *IRAR* is a potential target for prevention and/or attenuation of AKI.

## Introduction

Acute kidney injury (AKI) has emerged as a worldwide public health problem that is associated with a high risk of mortality, increased length of hospital stay, and development of chronic kidney disease [1–3]. In the past decades, the incidence of AKI has been progressively increasing, and unfortunately, no effective interventions have yet been developed to improve outcomes of established AKI, calling for a better understanding of its underlying pathophysiology.

Renal ischemia-reperfusion injury (IRI) is the most common cause of AKI in clinical settings. Sterile inflammation substantially contributes to the ischemia-reperfusion (IR) induced AKI, characterized by the rapid infiltration of inmmune cells, release of inflammatory cytokines and chemokines during ischemia and reperfusion [4]. Following ischemic injury, damage-associated molecular patterns (DAMPs) released from damaged kidney cells, mainly from tubular epithelial cells (TECs), initiate inflammation responses, and there is an inflammatory loop between TECs and immune cells during kidney IR, resulting in an inflammatory cascade that enhances kidney injury [5, 6]. Chemokines play an important role in the activation and migration of immune cells. Chemokines are chemotactic cytokines, including four families: CC, CXC, CX3C, and XC. Among these chemokine families, CXC chemokines, such as CXCL1 and CXCL2, mainly induce migration of polymorphonuclear leukocytes to the inflammatory tissue sites, and CC chemokines mainly attract mononuclear cells to the inflammatory sites [7, 8]. Emerging evidence has demonstrated that several chemokines are involved in inflammatory response in kidney IRI. For example, CXCL1 and CXCL2 are showed to be relevant to the neutrophil infiltration after IR, C–C motif chemokine ligand 2 (CCL2) and CX3CL1 are associated with the recruitment of monocytes /macrophages to the injury kidney [8, 9]. The role of chemokines in AKI needs to be further elucidated.

Long noncoding RNAs (lncRNAs) are typically defined as transcripts of >200 nucleotides in length without protein-coding potential, acting as key regulators in many physiological and pathological processes, including cell proliferation and differentiation, apoptosis, and inflammation [10, 11]. LncRNAs may interact with chromatin DNA, mRNA or protein, regulating mRNA expression and protein activity or stability [12, 13]. Dysregulation or dysfunction of lncRNAs has been shown to cause aberrant genes expression, and associated with a variety of human diseases including cardiovascular and neurological diseases, diabetes, and cancers [14–17]. Accumulating evidence indicates that lncRNAs are heavily involved in kidney diseases, including AKI and chronic kidney disease [16, 17]. LncRNA *PVT1* was identified by genome-wide association studies and contributed to end-stage renal disease in patients with type 2 diabetes mellitus [18]. LncRNAs *np_5318, np_17856 and Arid2-IR* were associated with progressive kidney inflammation and fibrosis in the mice following UUO [19, 20]. Lin et al. characterized the lncRNA landscape of proximal tubular epithelial cells in hypoxic and inflammatory conditions, and validated three lncRNAs (MIR210HG, linc-ATP13A4-8, and linc-KIAA1737-2) in human kidney tissue of AKI [21]. However, the underlying mechanisms of lncRNAs in the pathogenesis of renal IRI remain undefined.

In this study, we performed a genome-wide lncRNA microarray analysis of IR mice kidneys, and identified lncRNA *NR_003548*, which we have named ischemia-reperfusion-associated RNA (*IRAR*). It was the most highly upregulated lncRNA induced by IR in kidney, and functional experiments reveal that it plays an important role in the pathogenesis of renal IRI. Mechanistically, *IRAR* positively regulated expressions of chemokine CXCL1, CXCL2 and CCL2 in TECs, and knockdown of *IRAR* significantly reduced IR-induced immune cells infiltration, inflammatory factors production, and subsequently attenuated acute kidney injury, indicating *IRAR* as a potential therapeutical target for kidney disease.

## Materials and methods

### Animals and animal experiments

Male C57BL/6J mice were commercially obtained from the Animal Resource Center of Fudan University. CCL2^flox/flox^ mice (T001921, B6/J-Ccl2tm1Nju) on a C57BL/6J background were purchased from Nanjing Biomedical Research Institute of Nanjing University (Nanjing, China). Cdh16-cre mice (012237, B6.Cg-Tg(Cdh16 -cre)91Igr/J) were purchased from the Jackson Lab, which express Cre recombinase under the control of the mouse cadherin 16 (*Cdh16*) promoter, and the Cre recombinase is expressed in epithelial cells of the developing kidney and in the renal tubules of adult mice. CCL2^flox/flox^ mice were crossed with Cdh16-cre mice to produce renal tubular epithelial cell-specific CCL2-deficient mice, which are designated as TE-CCL2 KO. Homozygous pups were of normal body weight and size, and appeared to be healthy. Adult (8 to 10 weeks of age) mice were used in the animal experiments, and fed a standard normal diet with free access to rodent food and water. All animal procedures in this study were approved by the Institutional Animal Care and Use Committee of Fudan University.

Renal IRI was induced by bilateral renal pedicle clamping for 30 min followed by release for reperfusion, as described previously [22]. In sham-operated mice, renal pedicle were exposed but not clamped. The selective antagonist of CXCR2 SB225002 (N-(2-hydroxy-4-nitrophenyl) -N-(2-bromophenyl)urea) was used to evaluate the effect of CXCR2 inhibition on renal IRI. SB225002 was diluted in vehicle (0.9% NaCl solution containing 0.33% Tween-80). The mice received intraperitoneal administrations of SB225002 (50 μg in 200 μl) or vehicle 1 h prior and 1 h after renal IR, as previously described [23].

Cisplatin-induced AKI was established by a single intraperitoneal injection of cisplatin (20 mg/kg body weight) or vehicle (Saline), as previously described [24].

### In vivo knockdown of IRAR using antisense LNA™ longRNA GapmeR

For Locked nucleic acid (LNA)-GapmeR treatment, GapmeR *IRAR* (5′-GCATGGTGGAGG AGTT-3′) or Negative control (5’-GCTCCCTTCAATCCAA-3’) (Exiqon, Woburn, MA) were diluted in saline and injected intraperitoneally at a single dose of 20 mg/kg within 30 min before IR surgery, as described previously [25].

### Cell culture, transient transfection, and lentivirus transfection

Mouse renal tubular epithelial cells (mTECs) were purchased from Caltag Medsystems (Buckingham, UK), human proximal tubular epithelial cells (HK-2) were obtained from the American Type Culture Collection (Manassas, VA), which were cultured in a humidified atmosphere of 5% CO_2_ at 37 °C in Dulbecco’s modified Eagle’s medium supplemented with 10% fetal bovine serum. For hypoxia, a hypoxia incubator was used, and the cells were cultivated under the desired level of hypoxia (2% O_2_) for 24 h.

mTECs were transfected with GapmeR *IRAR* or Negative control oligonucleotides at final concentrations of 50 nM using Lipofectamine 3000 (Invitrogen) according to the manufacturer’s protocol. The LNA-GapmeR transfections were performed for 48 h. Then, the cells were treated with hypoxia as mentioned above. To silence C/EBP β expression, the synthesized C/EBP β siRNA oligonucleotides were transfected into mTECs using Lipofectamine 3000, and the scrambled oligonucleotides were used as negative control. The C/EBP β siRNA had the following sequence: sense: 5’-CCAUGGAAGUGGCCAACUUTT-3’; antisense: 5’-AAGUUGGCCACUUCCA UGGTT-3’. Forty-eight hours after the transfection, the cells were subjected to hypoxia.

Lentiviral vectors expressing lncRNA *IRAR* gene or enhanced green fluorescent protein (GFP) were constructed as previously described [26]. Lentiviral vector and packaging vectors (pLV-CMV-STAT5a, p8.9, pVSV-G) were transfected into the packaging cell line HEK293T using the transfection reagent RNAi-mate (Genepharma, Shanghai, China). The media containing lentiviruses were collected 72 h later, and incubated with mTECs. Overexpression efficiency were detected 72 h after infection.

### Microarray hybridization

Total RNA was isolated from kidneys of sham and IR mice (*n* = 3) using Trizol reagent (Invitrogen, Carlsbad, USA). Arraystar Mouse LncRNA Microarray V3.0 (KangChen Bio-technology Company, Shanghai, China) containing 35,923 lncRNA probes and 24,881 coding transcript probes was used to detect the global profiling of mouse lncRNAs and protein-coding transcripts. RNA labeling and array hybridization were performed according to the Agilent One-Color Microarray-Based Gene Expression Analysis protocol (Agilent Technology, USA). The hybridized arrays were scanned with the Agilent DNA Microarray Scanner (G2505C).

### Microarray data analysis

Raw data was extracted and the acquired array images were analyzed using Agilent Feature Extraction software (version 11.0.1.1). Quantile normalization and subsequent data processing were performed using GeneSpring GX v12.1 software package (Agilent Technologies, Santa Clara, CA, USA). Differentially expressed lncRNAs and mRNAs with statistical significance between the sham group and IR group were identified through fold-change filtering (≥2.0), multiple hypothesis testing (FDR < 0.05), and unpaired *t*-tests (*p* < 0.05). A volcano plot filtering was used to show differentially expressed lncRNAs, and differential mRNA expression profile between sham and IR mice was showed by Hierarchical clustering. Pathway analysis was based on KEGG (Kyoto Encyclopedia of Genes and Genomes) to identify the significant pathways of the differentially expressed mRNAs. The microarray data have been deposited in NCBI Gene Expression Omnibus and are provided at: https://www.ncbi.nlm.nih.gov/geo/query/acc.cgi?acc=GSE131454. The accession number is GSE131454.

### 5’ and 3’ rapid amplification of cDNA ends analysis

Rapid amplification of cDNA ends (RACE) experiments were performed using the GeneRacer^TM^ kit (Invitrogen) according to the manufacturer’s instructions. The gene-specific primers used for PCR were listed in Spplemental Table 1.

### Fluorescence in situ hybridization

RNA-FISH analysis was performed as previously described [27]. Briefly, mTECs were fixed in 4% formaldehyde and washed with PBS. Fixed cells were treated with 4% pepsase (2500–3000 U/mg) and then dehydrated. The air-dried cells were prehybridized with prehybridization buffer. Hybridization using *IRAR* probe (Table S1) was performed overnight at 37 °C. Then, slides were washed in 2 × SSC buffer, and counterstained with 4’-6’diamidino-2-phenylindole (DAPI) for 10 min. Images were recorded using a confocal microscope (Zeiss LSM 700, Germany). In addition, FISH was performed to detect co-expression of lncRNA-IRAR and CXCL1, CXCL2, CCL2 on kidney. Frozen sections were fixed in 4% paraformaldehyde, and treated with protease K (20 μg/ml) at 37 °C. After prehybridization, hybridization using *IRAR* probe was performed overnight. Slides were washed with SSC. Then, second hybridization using CXCL1, CXCL2, and CCL2 probes (Spplemental Table 1) was performed overnight at 37 °C, respectively.

### Nuclear and cytosolic fractions

In all, 1 × 10^7^ cells were collected and resuspended for separation of nuclear and cytosolic fractions. PARIS Kit (Life Technologies) was used according to the manufacturer’s instructions.

### RNA immunoprecipitation

RNA immunoprecipitation (RIP) was performed with the Magna RIP RNA-Binding Protein Immunoprecipitation Kit (Millipore, Burlington, MA) according to the manufacturer’s instructions. The anti-CXCL1 antibody (Invitrogen),anti-CXCL2 antibody (ab25130, Abcam), anti-CCL2 antibody (sc-52701, Santa Cruz Biotechnology) and control IgG were used for RIP. Co-precipitated RNAs were detected using real-time PCR. Total RNAs (input controls) and IgG controls were measured simultaneously.

### RNA pulldown

RNA pulldown was performed as previously described [28]. In brief, biotin-labled RNA of *IRAR* and the control transcripts were transcribed in vitro, treated with RNAse-free DNAse I and purified by RNeasy Mini Kit (QIAGEN, San Diego, CA). Three micrograms of biotinylated RNA was incubated with 1 mg of protein for 1 h at room temperature. Then, 60 μl of washed streptavidin agarose beads (Invitrogen) was added to each binding reaction, and the mixtures were incubated for 1 h at room temperature. The beads were washed and boiled in SDS buffer, the proteins that were pulled down were used for western blot.

### Chromatin immunoprecipitation

A chromatin immunoprecipitation (ChIP) assay was performed using the Pierce Agarose ChIP Kit (Thermo) according to the manufacturer’s instructions as described previously [29]. An anti-C/EBP β antibody (ab32358, Abcam) was utilized to immunoprecipitate crosslinked protein-DNA complexe, and anti-IgG antibody was used as a negative control. PCRs were performed with specific primers. The primers were listed in Supplemental Table 1.

### Coding noncoding co-expression network

The coding noncoding co-expression (CNC) network was constructed based on correlation analysis between the lncRNA *IRAR* and the differentially expressed mRNAs. The correlation coefficient was calculated between the normalized signal intensity of *IRAR* and the normalized differentially expressed mRNAs. The records with abs (pcc) ≥ 0.9, *p*-value ≤ 0.01 and FDR ≤ 0.01 were selected. The network was drawn using the bioinformatics software Cytoscape v2.8.3. Red node indicates lncRNA, and blue node indicates mRNA. Solid line indicates positive correlation, and dotted line indicates negative correlation.

### Luciferase assay

Mouse *IRAR* promoter was predicted from the National Center for Biotechnology Information (NCBI). Mutation within C/EBP β and *IRAR* binding site was generated using a KOD Plus mutagenesis kit (Toyobo) according to the manufacturer’s instruction. The pGL3 basic-lncRNA *IRAR* promoter plasmid or mutant plasmid were cotransfected with luciferase reporter plasmids into mTECs by Lipo2000. After transfection for 48 h, Firefly and Renilla activities in lysates were measured using Dual-Glo Luciferase Assay System (Promega, Madison, WI).

### Histopathological examinations and serum creatinine assay

Kidney sections were stained with hematoxylin and eosin and evaluated under light microscopy. Renal histopathology was evaluated by a nephropathologist blind to treatment assignment. Histologic injury scores were determined using scoring system, as described previously [29]. Serum creatinine was measured in 100 µl of serum using an automated analyzer (Vet test 8008, USA).

### Immunohistochemistry staining

Immunohistochemistry was performed as described previously [30]. The primary antibody was anti-CCL2 (ab8101, Abcam), and horseradish peroxidase-conjugated anti-rabbit IgG was used as secondary antibody.

### Immunofluorescence staining

Immunofluorescence staining was performed as described previously [31]. Anti-Gr-1 (BD Pharmingen Bioscience, San Diego, CA), anti-F4/80 (ab6640, Abcam), anti-CXCL1 (ab86436, Abcam), anti-CXCL2 (Novus, Littleton, CO), anti-CCL2 (ab8101, Abcam) and FITC-labeled Lotus tetragonolobus lectin (FL-1321, Vector Laboratories, Burlingame, CA) were used as primary antibodies for frozen sections of mouse kidney. Secondary Alexa Fluor 488–or Alexa Fluor 555–conjugated antibodies against rabbit or goat immunoglobulin (Invitrogen, Carlsbad, CA) were used to visualize antigen-antibody complexes. 4’,6-diamidino-2 -phenylindole (DAPI) was used for nuclear staining. Images were obtained using a confocal microscope (Zeiss LSM 700, Germany)..

### Enzyme-linked immunosorbent assay (ELISA)

Concentrations of IL-6, CXCL1, CXCL2 and CCL2 in blood and tissue homogenate were examined by commercially available ELISA kit (IL-6: R&D Systems Inc., Minneapolis, USA; CXCL1: ab216951, Abcam; CXCL2: ab204517, Abcam; CCL2: ab100721, Abcam), according to the manufacturer’s protocol.

### Flow cytometry

To examine the infiltrated leukocytes in kidney, single-cell suspensions were prepared as previously described [32], and stained using the the following fluorochrome-labeled antibodies: anti-CD45 (PE-CY5), CD11b (FITC), Ly6G (APC), and F4/80 (eFluor 450) (eBioscience, CA, USA). Flow cytometry was performed on a FACSCalibur (Becton Dickinson, Heidelberg, Germany), analyzed using FlowJo 10.0 software. Single-cell suspensions of kidney were prepared.

The AnnexinV-FITC/propidium iodide (PI) staining kit was applied to assess cell apoptosis using a flow cytometer (Accuri C6, BD) according to the manufacturer’s instructions. Quadrant statistics on AnnexinV-positive/PI-negative, double-positive cells were performed.

### Real-time polymerase chain reaction (PCR)

Total RNA was extracted from mTECs and kidney tissue using Trizol reagent (Invitrogen, Carlsbad, CA). After reverse transcription to cDNA (PrimeScript RT reagent Kit; TaKaRa, Kyoto, Japan), real-time polymerase chain reaction (PCR) was performed using SYBR^®^ Premix Ex Taq^TM^ II (DRR081A, TaKaRa). The specific primers (Sangon, Shanghai, China) were listed in Supplemental Table 1. β-actin mRNA were used as endogenous control. Relative changes in mRNA expression were calculated using the 2^–ΔΔCt^ method.

### Western blot

Western blot was performed as previously described [30]. The primary antibodies were used as follows: anti-CXCL1 (ab86436, Abcam), anti-CXCL2 (ab25130, Abcam), anti-CCL2 (ab8101, Abcam), anti-C/EBP β antibody (ab32358, Abcam), and anti-β-actin (sc-47778, Santa Cruz Biotechnology).

### Statistical analysis

Data are expressed as mean ± SEM. The differences between two groups were analyzed by two-tailed, unpaired *t*-tests. Multiple comparisons were examined using one-way ANOVA followed by Bonferroni posttest. Statistical significance was set at a *p*-value < 0.05.

## Results

### Newly identified lncRNA involved in renal ischemia-reperfusion injury

**Fig. 1:**
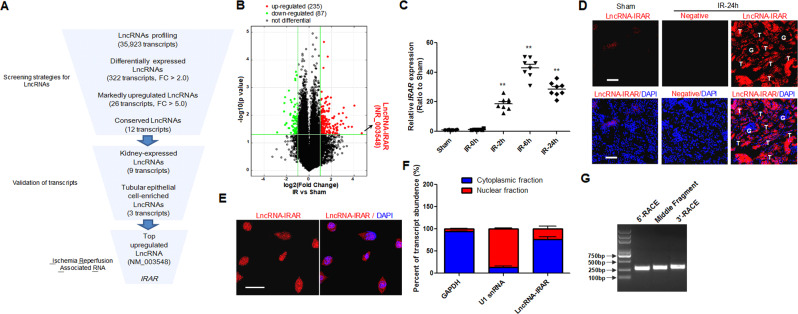
*IRAR* is induced by ischemia reperfusion (IR) in mice. **A** Screening strategy of IR-induced murine lncRNAs derived from microarray profiling. **B** A volcano plot showed the differentially expressed lncRNAs in IR group compared to Sham group. Red and green dots represent upregulated and downregulated lncRNAs in kidneys 24 h after IR, respectively (fold-change ≥ 2.0 and *p*-value ≤ 0.05). Black dots represent the genes that were not differentially expressed. *IRAR* is highlighted in red. **C** Time course of *IRAR* expression. Data represent mean ± SEM. *n* = 6-8, **p < 0.01 versus Sham group. **D** RNA fluorescence in situ hybridization (FISH) assay of *IRAR* in the mouse kidney samples at 24 h after IR. Scale bar, 100 μm. **E** FISH assay of *IRAR* in the hypoxia-treated mTECs. Scale bar, 25 μm. **F** The expression level of *IRAR* in cytoplasm and nuclei of hypoxia-treated mTECs. GAPDH (cytoplasm retained) and U1 (nuclear retained) were used as controls. **G** 3′ and 5′ rapid amplification of cDNA ends (RACE). Agarose gel electrophoresis of PCR products from the 5′-RACE and 3′-RACE procedure.

To identify the potential involvement of lncRNAs in kidney during IR, we performed a global profiling of lncRNAs in the kidneys from mice undergoing IRI or sham surgery. The microarray contained 35,923 lncRNA probes, and most lncRNAs represented on the array (20,045 lncRNAs) were detected. Bioinformatic filtering of the array dataset was performed as showed in Fig. 1a. In total, 322 lncRNAs were identified as being differentially expressed in kidneys after IR (fold-change: log2|FC | > 2 or <2, *p* < 0.05), 235 lncRNAs were upregulated, whereas 87 lncRNAs were downregulated (Fig. 1b). Next, we selected 22 conserved and highly dysregulated (12 upregulated with log2|FC | > 5, and 10 downregulated with log2|FC | > 3) candidates for validation. Quantitative real-time polymerase chain reaction (qRT-PCR) was performed to analyze the expression of these 22 lncRNAs in the mouse kidneys 24 h after IR. The expression of most lncRNAs (9 of 12 upregulated lncRNAs, 10 of 12 downregulated lncRNAs) was consistent with the microarray data (Supplemental Fig. 1A, B). Next, we detected those 9 upregulated transcripts in hypoxia-treated primary TECs to identify TEC-enriched lncRNAs, and found three lncRNAs, namely *NR_003548*, *NR_045935* and *AK131807*, were highly expressed in TECs (Supplemental Fig. 1C). Among these TEC-enriched transcripts, *NR_003548* was the most abundant lncRNA both in ischemia-reperfusion kidney and hypoxia-treated TECs. Finally, *NR_003548* was selected for further analysis, and it was designated as lncRNA-IRAR.

We measured *IRAR* expression in kidneys during IR, and found that *IRAR* was upregulated in a time-dependent manner, and peaked at 6 h after IR (Fig. 1C). In situ hybridization analysis confirmed the upregulation of lncRNA-IRAR in the mouse kidney samples at 24 h after IR (Fig. 1D), and *IRAR* was predominantly located in the renal tubules. Subcellular fractionation assays revealed that *IRAR* was present in both nuclei and cytoplasm of TECs, and the expression of *IRAR* in the cytoplasm was higher than that in the nucleus (Fig. 1E, F). Then, we analyzed full-length sequence of *IRAR* in mouse TECs using 3′ and 5′ RACE, confirming that *IRAR* is a 658-nt polyadenylated transcript (Fig. 1G and Supplemental Fig. 2). The validated sequence strongly correlates to the sequence information of transcript *NR_003548* annotated by NCBI. The coding potential prediction software (http://www.noncode.org) indicates that *IRAR* is a noncoding RNA with CNCI Score -0.0294912.

### Chemokines production and immune cells infiltration in kidney during renal ischemia reperfusion

**Fig. 2:**
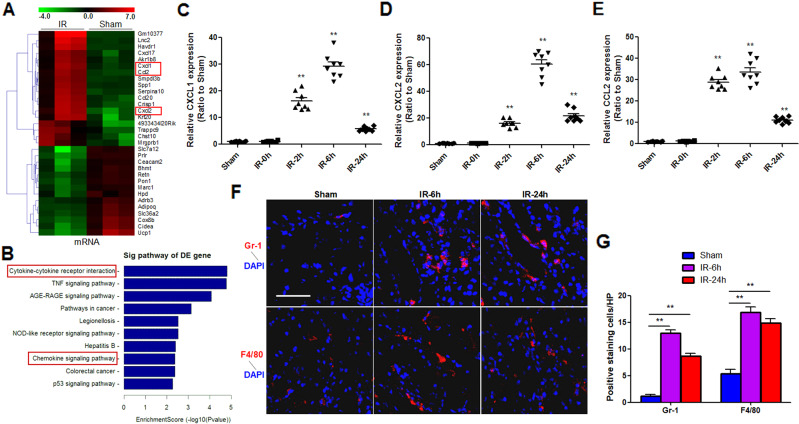
Ischemia-reperfusion (IR) induces production of chemokines and infiltration of immune cells. **A** Hierarchical clustering analysis of the differentially expressed mRNAs in IR group compared to Sham group (green, low; red, high). Three significantly upregulated mRNAs: CXCL1, CXCL2, and CCL2 (fold-change > 10) were highlighted in red box. **B** KEGG pathway enrichment analysis for upregulated mRNAs. **C**–**E** Time course of chemokine CXCL1 (**C**), CXCL2 (**D**), and CCL2 (**E**) expression. Data represent mean ± SEM. *n* = 6–8. **p < 0.01 versus Sham group. **F** Immunofluorescence staining of Gr-1 and F4/80 in the kidney 6 h and 24 h after renal IR. Gr-1 was used as a marker of neutrophils, and F4/80 was used as a marker of macrophages. Scale bar, 100 μm. **G** Quantification of Gr-1-positive neutrophils and F4/80-positive macrophages in the kidneys. Data represent mean ± SEM. *n* = 6. **p < 0.01.

To characterize the expression pattern of mRNAs in IR-induced kidney injury, we performed mRNA microarray analysis for the total RNAs of kidneys from mice undergoing IRI or sham surgery. The results showed that 192 mRNAs were upregulated and 75 mRNAs were downregulated after renal IR (fold-change ≥ 2; *p* < 0.05), among which several chemokines such as CXCL1, CXCL2, CCL2, were significantly upregulated (fold-change > 10) (Fig. 2A). Pathway analysis indicated that genes associated with cytokines and chemokines signaling pathways were highly upregulated during renal IR (Fig. 2B). Next, we detected the expression profiles of CXCL1, CXCL2, CCL2 mRNAs during renal IR and found that they were all increased in a time-dependent manner, and peaked at 6 h after IR, which was consistent with the *IRAR* expression. (Fig. 2C–E). Immunofluorescence staining showed that infiltration of Gr-1-positive neutrophil and F4/80-positive macrophage was significantly increased in the kidneys 6 and 24 h after IR, responding to the increased chemokines (Fig. 2F, G). Flow cytometry was used to detect the recruitment of neutrophils and macrophages in kidneys, which also showed increased number of infiltrated neutrophils and macrophages in kidneys after IR (Supplemental Fig. 3).

### Knockdown of *IRAR* attenuates inflammation and acute kidney injury

**Fig. 3:**
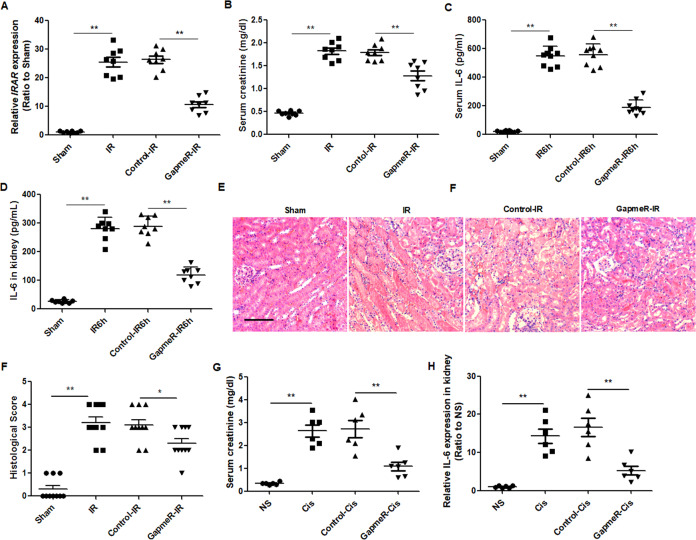
Inhibition of *IRAR* in vivo attenuates inflammation and acute kidney injury. The GapmeR *IRAR* was injected into mice 1 h before IR operaton. GapmeR-control was a scrambled sequence. **A** Quantitative RT-PCR analysis of *IRAR*. **B** Serum creatinine 24 h after renal IR. **C**, **D** ELISA of IL-6 in circulation and in the kidney 6 h after renal IR. **E** Hematoxylin–eosin staining for kidneys. Original magnification, ×200. Arrow indicates renal tubular epithelial cell necrosis. Scale bar, 100 μm. **F** Tubular damage score in renal cortical tissues. **G** Serum creatinine 72 h after cisplatin injection. GapmeR IRAR or Negative control was injected into mice via tail vein 1 h before cisplatin injection. **H** qRT-PCR analysis of IL-6 in kidney. Data represent mean ± SEM. *n* = 6–8. **p < 0.01.

To investigate whether *IRAR* affected renal IRI in vivo, we knocked down *IRAR* in the mouse kidney with GapmeRs-antisense oligonucleotides. The GapmeR *IRAR* was injected into mice via tail vein 1 h before IR operation, and *IRAR* was significantly downregulated in the mouse kidneys 24 h after renal IR (Fig. 3A). We observed an obvious reduction in serum creatinine in the mice injected with GapmeR *IRAR* when compared with the GapmeR-control injected mice (Fig. 3B). Furthermore, the level of proinflammatory cytokine IL-6 in serum and renal tissue was significantly reduced 6 h after IR in mice treated with GapmeR *IRAR* (Fig. 3C, D). Morphologic changes were evaluated using histopathologic scoring of cortical tubular damage, and the results showed that morphologic damage was also attenuated in the mice treated with GapmeR *IRAR* (Fig. 3E, F). In addition, we investigated the effects of *IRAR* on AKI in another mouse model. Cisplatin-induced AKI was induced by a single dose of cisplatin (20 mg/kg i. p.) to mice. GapmeR *IRAR* or Negative control was injected into mice via tail vein 1 h before cisplatin injection. Consistent with the results of IR-induced AKI, the mice received GapmeR *IRAR* exhibited lower serum creatinine level at 72 h after cisplatin treatment than the mice treated with Negative control. Moreover, the level of IL-6 in kidneys was significantly reduced in the GapmeR *IRAR* pretreatment mice when compared with GapmeR-control injected mice. These results suggest that silencing of *IRAR* protected against cisplatin-induced AKI (Fig. 3G, H).

### Knockdown of *IRAR* reduces chemokines production and immune cells infiltration during renal ischemia reperfusion

**Fig. 4:**
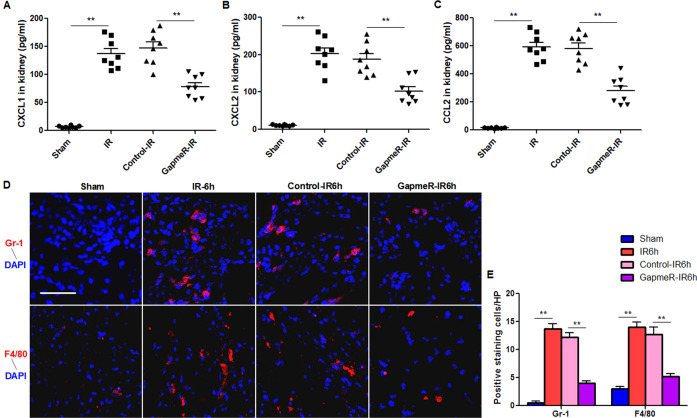
Inhibition of *IRAR* reduces ischemia-reperfusion (IR)-induced production of chemokines and infiltration of immune cells. **A**–**C** ELISA of CXCL1 (**A**), CXCL2 (**B**), CCL2 (**C**) in the kidney 24 h after renal IR (*n* = 6–8). **D** Immunofluorescence staining of Gr-1 and F4/80 in the kidney 6 h after renal IR. Gr-1 was used as a marker of neutrophils and F4/80 was used as a marker of macrophages. Scale bar, 100 μm (*n* = 6–8). **E** Quantification of Gr-1-positive neutrophils and F4/80-positive macrophages in the kidneys. Data represent mean ± SEM. *n* = 4. *p < 0.05, **p < 0.01.

We investigated the effects of silencing of *IRAR* on chemokines production and immune cells infiltration during renal IR. The results indicated that the levels of chemokine CXCL1, CXCL2, CCL2 were significantly reduced 24 h after IR in mice treated with GapmeR *IRAR* (Fig. 4A–C) when compared with the GapmeR-control treated mice. Moreover, immune cells infiltration in kidneys, including neutrophils and macrophages, were reduced 6 h after IR in GapmeR *IRAR* mice (Fig. 4D, E).

### *IRAR* regulates expressions of CXCL1, CXCL2, and CCL2 in tubular epithelial cells

**Fig. 5:**
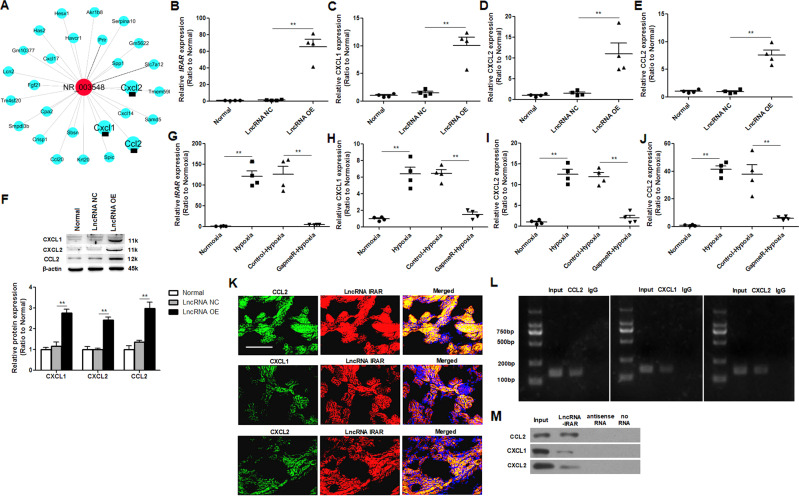
*IRAR* regulates chemokine expression. **A** Co -expression network of *IRAR* and differentially expressed mRNAs. The solid line indicates positive regulation and the dotted line indicates negative regulation. **B** qRT-PCR analysis of *IRAR* in mTECs transfected with *IRAR* overexpressing lentivirus (LncRNA-OE) or Negative lentivirus (LncRNA-NC). **C**–**E** qRT-PCR analysis of CXCL1 (**C**), CXCL2 (**D**), CCL2 (**E**) in mTECs transfected with *IRAR* overexpressing lentivirus or Negative lentivirus. **F** Western blot analysis of CXCL1, CXCL2 and CCL2 in mTECs transfected with *IRAR* overexpressing lentivirus or Negative lentivirus.. Data are means from three independent experiments. **G**–**J** qRT-PCR analysis of *IRAR* (**G**), CXCL1 (**H**), CXCL2 (**I**) and CCL2 (**J**) in mTECs. mTECs were transfected with GapmeR *IRAR* (GapmeR-Hypoxia) or Negative control (Control-Hypoxia) for 48 h, then treated with hypoxia. **K** RNA fluorescence in situ hybridization (FISH) assays indicated co-expression between *IRAR* and CXCL1, CXCL2, CCL2 in the kidney at 24 h after ischemia reperfusion. Scale bar, 100 μm. **L** RNA immunoprecipitation (RIP) experiments were performed using antibodies against CXCL1, CXCL2 and CCL2. RIP enrichment was determined relative to the input controls. **M** RNA pulldown assays were performed in tubular epithelia cells to examine the association of *IRAR* with CXCL1, CXCL2 and CCL2. Data represent mean ± SEM. *n* = 4. **p < 0.01.

Coding noncoding co-expression (CNC) analysis was performed to construct co-expression networks of differentially expressed lncRNAs and mRNAs. Through the construction of *IRAR*-mRNAs co-expression network, we found that *IRAR* positively regulated the expressions of chemokine CXCL1, CXCL2, CCL2 (Fig. 5A). Next, we investigated the effect of *IRAR* overexpression on CXCL1, CXCL2, CCL2 expression in mTECs. *IRAR* was overexpressed by transfection with *IRAR* overexpressing lentivirus. Negative lentivirus was as control. The results revealed that *IRAR* overexpression induced CCL2, CXCL1, CXCL2 expression both in mRNA and protein levels (Fig. 5B–F). Then, we investigated the effect of silencing of *IRAR* on CXCL1, CXCL2, CCL2 expression in mTECs treated with hypoxia. In mTECs, transfection with GapmeR *IRAR* led to a significant reduction of the *IRAR* level (Fig. 5G), and silencing of *IRAR* resulted in significant downregulation of CCL2, CXCL1 and CXCL2 transcription (Fig. 5H–J).

Gene co-localization analysis is thought to play an important role in determining gene expression patterns and co-regulation. RNA-fluorescence in situ hybridization revealed that *IRAR* colocalized with CCL2, CXCL1 and CXCL2 transcript in the kidney 24 h after IR (Fig. 5K). To examine whether *IRAR* associated with CCL2, CXCL1, or CXCL2, we performed RNA immunoprecipitation (RIP) with antibodies against CCL2, CXCL1, CXCL2 from the cell extracts of hypoxia-treated mTECs, and found significant enrichment of *IRAR* with the antibodies CCL2, CXCL1, CXCL2, respectively, compared with the IgG control antibody (Fig. 5L). Next, we performed a RNA pulldown assay to validate the association between *IRAR* and CCL2, CXCL1, CXCL2 in mTECs. Biotinylated *IRAR* or antisense RNA was incubated with the extracts of mTECs, western blot analysis confirmed that *IRAR* physically associated with CCL2, CXCL1, CXCL2 in vitro (Fig. 5M). Collectively, these data suggested that *IRAR* regulated expressions of CCL2, CXCL1, and CXCL2 in mTECs.

### *IRAR* increases proinflammatory cytokine production and apoptosis in human proximal tubular epithelial cells

We performed sequence homology analysis and found that the murine lncRNA *IRAR* has a human ortholog RNA in HG19, referred to as uc001fbf.3, which is located on human chromosome 1 (chr1:153066013-153066247). We used human proximal tubular epithelial cells (HK-2) for its human relevance and thus performed a portion of in vitro studies in this cell line. We investigated the effects of *IRAR* overexpression on production of proinflammatory cytokines, including chemokines and proinflammatory factor IL-6, and apoptosis in HK-2 cells under hypoxia. *IRAR* was overexpressed in HK-2 cells by transfection with *IRAR* overexpressing lentivirus. The results revealed that *IRAR* overexpression induced CCL2, CXCL1, CXCL2 expression (Supplemental Fig. 4A–D), promoted proinflammatory factor IL-6 production (Supplemental Fig. 4E), and increased apoptosis (Supplemental Fig. 4F).

We also investigated the effects of silencing of *IRAR* on production of proinflammatory cytokines and apoptosis in HK-2 cells under hypoxia. The results showed that GapmeR-mediated silencing of *IRAR* in HK-2 cells significantly downregulated CCL2, CXCL1 and CXCL2 expression (Supplemental Fig. 5A–D), inhibited IL-6 production (Supplemental Fig. 5E), and decreased apoptosis (Supplemental Fig. 5F).

### Inhibition of CXC chemokine receptor 2 attenuates IR-induced neutrofils infiltration and proinflammatory factor production

**Fig. 6:**
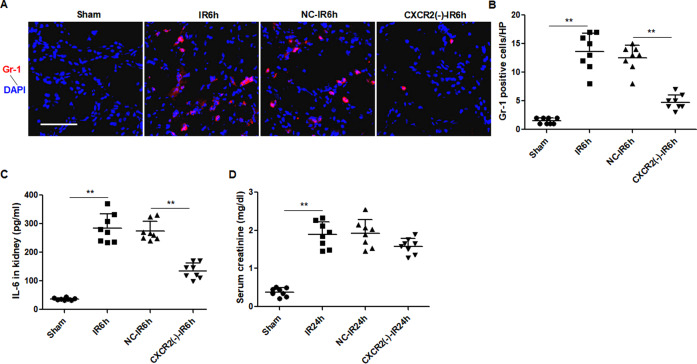
Inhibition of CXC chemokine receptor 2 (CXCR2) attenuates ischemia-reperfusion (IR)-induced inflammation. The mice received intraperitoneal administrations of antagonist of CXCR2 (CXCR2(-)-IR) or vehicle (NC-IR) 1 h prior and 1 h after renal IR. **A** Immunofluorescence staining of Gr-1 in the kidney 6 h after renal IR. Scale bar, 100 μm. **B** Quantification of Gr-1-positive neutrophils. **C** Concentration of serum creatinine 24 h after renal IR. **D** ELISA of IL-6 in the kidney 6 h after renal IR. Data represent mean ± SEM. *n* = 6–8. **p < 0.01.

CXCL1 and CXCL2 are thought to exert their effects through the CXC chemokine receptor 2 (CXCR2), promotes migration of CXCR2^+^ leukocytes, especially neutrophils, to the local sites, contributing to inflammation [33]. IR induced upregulation of CXCL1 and CXCL2 in the kidney, which were mainly located in renal tubular epithelia (Supplemental Fig. 5). To investigate the effects of CXCL1 and CXCL2 on IR-induced inflammation and renal dysfunction, we inhibited the activity of their receptor CXCR2 using selective antagonist of CXCR2 in IR mice. The results showed that CXCR2 antagonist significantly decreased neutrophils infiltration at 6 h after renal IR, compared to the contol reagent (Fig. 6A, B). Consistently, the production of proinflammatory factor IL-6 was markedly reduced in kidneys of CXCR2 antagonist-treated mice (Fig. 6C). However, compared with the control reagent-treated mice, mice treated with CXCR2 antagonist did not show an overt decrease in serum creatinine 24 h after IR (Fig. 6D).

### Tubule-specific ablation of CCL2 ameliorates IR-induced macrophages infiltration and renal dysfunction

**Fig. 7:**
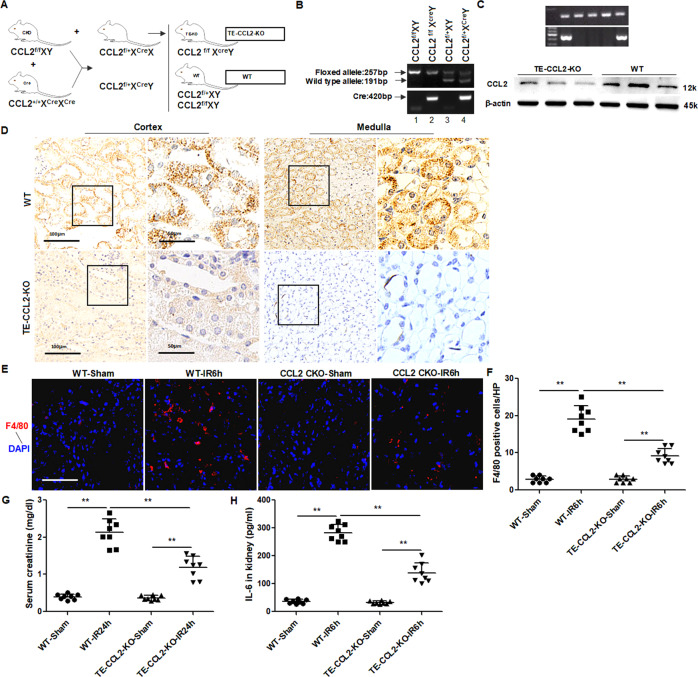
Tubule-specific ablation of CCL2 reduces macrophages infiltration and kidndy injury. **A** Breeding protocol for generating conditional tubular epithelial cell knockout mice (TE-CCL2-KO). **B** Representative gel images of genotyping. DNA was extracted from mouse tail and amplified, wild-type and floxed alleles of CCL2 and Cdh16-cre allele were detected. Lane 1 showed the genotyping of the wild-type mice used in this study (CCL2^fl/fl^), lane 2 showed the genotyping of the tubule-specific CCL2 knockout mice (CCL2^fl/fl^Cre, designated as TE-CCL2-KO). **C** Western blot analysis indicated a substantial reduction of renal CCL2 expression in TE-CCL2-KO mice 24 h after renal IR. **D** Representative images showed CCL2 staining in renal cortical and medullar regions of the WT and TE-CCL2-KO mice at 24 h after IR. Scale bar, 50 or 100 μm. **E** Immunofluorescence staining of F4/80 in the kidney 6 h after renal IR. Scale bar, 100 μm. **F** Quantification of F4/80-positive cells. **G** Concentration of serum creatinine 24 h after renal IR. **H** ELISA of IL-6 in the kidney 6 h after renal IR. Data represent mean ± SEM. *n* = 6–8. *p < 0.05, **p < 0.01.

CCL2, also referred to as monocyte chemotactic protein-1 (MCP-1), is a chemokine that can stimulate the release of monocytes from bone marrow and activate the migration of monocytes and macrophages to the local sites. CCL2 was mainly secreted from proximal tubular epithelial cells during IR (Fig. 5E, F, J and Supplemental Fig. 5). To investigate the pathologic role of tubular CCL2, we initially constructed a conditional knockout mouse model, in which CCL2 was deleted specifically from renal tubules. The breeding protocol was shown in Fig. 7A. We crossed CCL2^flox/flox^ mice with Cdh16-cre mice, in which Cre recombinase is expressed in renal tubules (http:// jaxmice.jax.org/strain/012237.html), yielding a mouse in which the TECs are deficient in CCL2, designated as TE-CCL2 KO.

To verify the genotypes, we performed PCR for each mouse. The genotype of TE-CCL2-KO mice was indicated by amplification of the 257-bp fragment of the floxed allele, not the 191-bp fragment of the wild-type (WT) allele, and amplification of the 420-bp fragment of the Cre gene (Fig. 7B). At the protein level, we compared CCL2 expression in kidneys of TE-CCL2-KO and WT mice after renal IRI. We found that CCL2 expression was significantly reduced in TE-CCL2-KO mice compared to the WT mice (Fig. 7C). Immunohistochemical staining for CCL2 also showed a tubule-specific reduction of CCL2 protein in the kidneys of TE-CCL2-KO mice (Fig. 7D), which verified tubular CCL2 ablation in the conditional model. TE-CCL2-KO mice and WT littermates were subjected to renal IR or sham procedure. The results showed that 30 minutes of renal IR induced severe AKI in WT mice, but moderate AKI in TE-CCL2-KO mice. In TE-CCL2-KO mice, macrophages infiltration was markedly reduced 6 h after renal IR (Fig. 7E, F), concentration of serum creatinine was lower at 24 h after renal IR (Fig. 7G), and IL-16 level was obviously decreased in the kidney (Fig. 7H), when compared with WT mice.

### Increased expression of *IRAR* is induced by transcription factor CCAAT enhancer binding protein beta (C/EBP β)

**Fig. 8:**
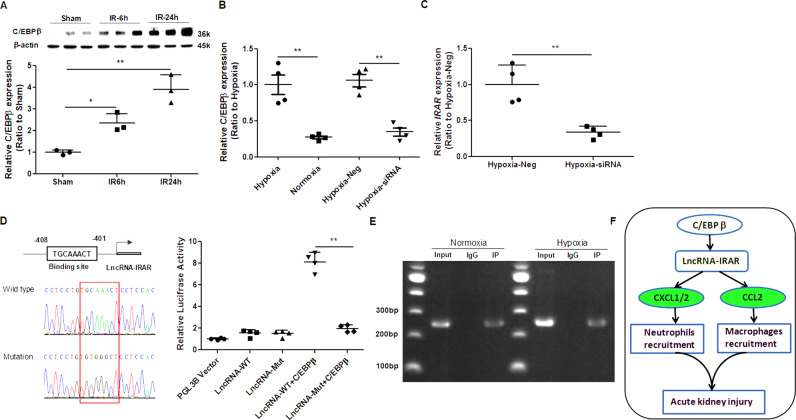
*IRAR* is regulated by the transcription factor C/EBP β during renal ischemia reperfusion. **A** Western blot analysis of C/EBP β during renal ischemia reperfusion. **B** qRT-PCR analysis of C/EBP β. mTECs were transfected with C/EBP β siRNA (Hypoxia-siRNA) or negative control oligonucleotides (Hypoxia-Neg), and then subjected to hypoxia. **C** qRT-PCR analysis of *IRAR* in hypoxia-treated mTECs transfected with C/EBP β siRNA or negative control. **D** Luciferase reporter assays were performed to determine the C/EBP β binding on the *IRAR* promoter region. Prediction and mutation of C/EBP β-binding site in the *IRAR* promoter region (left). Luciferase activity was shown as relative luciferase activity normalized to Renilla activity (right). **E** ChIP assays were performed to detect C/EBP β occupancy in the *IRAR* promoter region. IgG was as negative control. **F** Schematic representation of lncRNA-IRAR-mediated renal ischemia-reperfusion injury. Data represent mean ± SEM. *n* = 4. *p < 0.05, **p < 0.01.

To explore the mechanism of *IRAR* upregulation in kidney during IR, we analyzed promoter region of *IRAR* and predicted all transcription factors containing potential binding sites on *IRAR* promoter from Esenmble Database (Supplemental Fig. 6). Among these transcription factors, C/EBP β is an important regulator in immune response and inflammatory cascade, which is highly expressed in kidney [34]. We examined the expression of C/EBP β in the kidney during IR, and found that C/EBP β was significantly upregulated (Fig. 8A). To further investigate the correlation between *IRAR* and C/EBP β, we silenced C/EBP β expression with C/EBP β siRNA transfection in mTECs under hypoxia. Upon C/EBP β inhibition, *IRAR* expression was significantly downregulated (Fig. 8B, C). Using online bioinformatical software JASPAR (http://jaspar.genereg.net/cgi-bin/jaspar_db.pl), we found a potential binding site for C/EBP β on *IRAR* promoter (Fig. 8D, left). To confirm *IRAR* was a transcriptional target of C/EBP β, dual-luciferase reporter assay was performed, and the *IRAR* promoter containing the wild-type or mutated binding site was fused to a luciferase reporter vector. The results revealed that the activity of the luciferase reporter fusing wild-type promoter was significantly increased, whereas the activity of the luciferase reporter fusing mutated promoter with mutation of this site did not alter (Fig. 8D, right). Moreover, chromatin immunoprecipitation (ChIP) assay confirmed the C/EBP β was capable of binding to the *IRAR* promoter directly (Fig. 8E). Collectively, these results suggest that *IRAR* is regulated by C/EBP β.

## Discussion

The current study represents the first detailed report, to our knowledge, on the dysregulation of lncRNAs in kidney following IR, and identifies the lncRNA *IRAR* to be a crucial regulator of chemokine expression in TECs, promoting inflammatory response, and leading to a loss of renal function after IR (Fig. 8F). Silencing of *IRAR* in vivo significantly attenuated ischemic AKI by downregulation of chemokine CXCL1, CXCL2, and CCL2 expression in TECs, inhibition of inflammatory cells recruitment post kidney IR.

Previous studies have led to important advances in our understanding of the pathogenesis of renal IRI, emphasizing the roles of chemokines in this pathophysiological process [8, 9]. Chemokines are key players in the migration of chemokine receptor-expressing inflammatory cells to the diseased sites. The influx of the different types of leukocytes correlates with the upregulation of certain chemokines. Ferreira et al. characterized the spatial transcriptomic signature of kidney in two murine AKI models: IRI and sepsis-induced AKI, identifying two chemotactic factors *Atf3* and *Mdk*c, colocalized between immune and epithelial cells, which could induce neutrophil and macrophage infiltration respectively [35]. Sears et al. demonstrated that CCL2 was a marker of cisplatin-induced kidney injury, was associated with an increase in infiltrating renal macrophages and monocytes in C57BL/6 mice [36]. In this study, we demonstrated that both CC and CXC chemokines are highly upregulated during renal IR, including CCL2, CXCL1, and CXCL2, which induced macrophages and neutrophils infiltration, respectively. The functions of these chemokines and their receptors have already been investigated. Increasing studies in transgenic mice or blockade of chemokine signaling have demonstrated their roles of anti-inflammation or renoprotection in several expremental models of kidney injury. In a mouse model of streptozotocin-induced diabetic nephropathy, CCL2/MCP-1 deficiency suppressed renal injury by markedly reducing interstitial macrophages accumulation [37]. Genetic or pharmacological blockade of CXCL1-CXCR2 signaling reduced production of proinflammatory cytokines and infiltration of neutrophils in the kidneys following cisplatin treatment [38]. Consistent with these findings, here, we also found that administration of CXCR2 (receptor of CXCL1 and CXCL2) antagonists before renal ischemia reduced neutrophils infiltration and inflammatory factor production in kidney after IR. Moreover, deficiency of CCL2 could diminish macrophages infiltration and ameliorated renal function in IR mice.

Recently, many lncRNAs have been reported to be involved in inflammation and kidney disease, including AKI and chronic kidney disease [39–41]. Yu et al. reported hypoxia-induced lncRNAs in renal tubular cells, and identified *PRINS* as a hypoxia-induced factor-1α dependent lncRNA, which was significantly upregulated under hypoxic condition, interacted with chemokines, involved in the process of AKI [39]. Zhao et al. demonstrated that *DANCR* was decreased in HK-2 cells treated with lipopolysaccharide. Served as a sponge for miR-214, *DANCR* suppressed cytokine production in tubular epithelial cells, and attenuated lipopolysaccharide -induced AKI [40]. In chronic kidney diseases, *lRNA9884* was found to enhance the promoter activity of MCP-1, and promoted renal inflammation-driven diabetic kidney injury by upregulating MCP-1 expression in mice [41]. In the present study, we identified a novel inflammation-related lncRNA, which promoted ischemic AKI via regulating expression of chemokines. Besides regulating CXCL1, CXCL2, and CCL2 expression, it is likely that *IRAR* also influence expression of other chemokines and proinflammatory cytokines that are involved in inflammatory response, according to the gene co-expression pattern (Fig. 5A). Further studies will advance our understanding of this lncRNA in regulation of inflammation and renal injury.

In conclusion, we have reported a genome-wide expression profile of lncRNAs after renal IRI, highlighting that *IRAR* is a proinflammatory lncRNA, and in vivo targeting of *IRAR* could be beneficial in ischemic AKI. Our results support the idea that lncRNAs, such as *IRAR*, play criticial roles in inflammation, and suggest that *IRAR* is a potential target for prevention and/or attenuation of AKI.

## Supplementary information

Supplemental materials

Supplemental Fig. 1

Supplemental Fig. 2

Supplemental Fig. 3

Supplemental Fig. 4

Supplemental Fig. 5

Supplemental Fig. 6

Supplemental Fig. 7

full and uncropped western blots

checklist

## Data Availability

The data in the current study are available at: https://www.ncbi.nlm.nih.gov/geo/*query/ac c.cgi?acc* = *GSE131454* through the accession number GSE131454 and the corresponding author on request.
