# Supplementary material for: LncRNA IRAR regulates chemokines production in tubular epithelial cells thus promoting kidney ischemia-reperfusion injury
Source: Cell Death Dis. 2022 Jun 22;13(6):562. doi: 10.1038/s41419-022-05018-x (PMC9217935; doi:10.1038/s41419-022-05018-x)
Supplement: Supplementary file 1 — Supplemental materials [file 41419_2022_5018_MOESM1_ESM.docx]

**Supplemental material**

**Supplemental Table 1.** Oligonucleotide primers used in this study

| **RT-PCR primer (LncRNA)**  NR_003548 | forward | **Primer sequence**  5′-TACCAGATGTTCAGAGGAAAGAC-3′ |
| --- | --- | --- |
| humanlincRNA2416+  mouselincRNA1453  NR_045935  AK140152  ENSMUST00000162322  uc011zab.2  ENSMUST00000181062  ENSMUST00000154124  ENSMUST00000138787  AK131807  ENSMUST00000130656  TCONS_00013582  AK034039  TCONS_00013588  ENSMUST00000181103  ENSMUST00000161085  AK083073  ENSMUST00000172432  ENSMUST00000157020  AK140001  ENSMUST00000145428  **RT-PCR primer (mRNA)**  CXCL1  CXCL2  CCL2  C/EBP β  IL-6  β-actin  **RACE**  5′RACE-*IRAR*  3′RACE-*IRAR*  **CHIP**  *IRAR*  Nagetive control  **In situ hybridization**  *IRAR*  CXCL1  CXCL2  CCL2 | reverse  forward  reverse  forward  reverse  forward  reverse  forward  reverse  forward  reverse  forward  reverse  forward  reverse  forward  reverse  forward  reverse  forward  reverse  forward  reverse  forward  reverse  forward  reverse  forward  reverse  forward  reverse  forward  reverse  forward  reverse  forward  reverse  forward  reverse  forward  reverse  forward  reverse  forward  reverse  forward  reverse  forward  reverse  forward  reverse  forward  revers  forward  reverse    forward  reverse  forward  reverse | 5′-ACTGTGAAGGCACTGAGGGA-3′  5’-GAGTCCAAGAGTATTGTGCCA-3’  5’-TCCTCCTCCTCCATTCGTTA-3’  5’-CTCTGCTTCTGTTTCTTTCCTC-3’  5’-CAGTGTTATCACAAGTCAGTCCC-3’  5’-GGAGCAGAGCAATCAGCAGT-3’  5’-CGGAGTCACATAGCCAGAAAT-3’  5`-TGGTGTAAGCATCTGGGTAGTC-3`  5`-CCTGGGCCTTTCAGGAATA-3`  5’-ACCTCCTGCGTTGTGAAGAC-3’  5’-GATAACAGTGTGAAGGAAGGCATC-3’  5’-TGAGACCGTTCACAGCACTAC-3’  5’-GACCAGTCATAAGCCCAGGAG-3’  5’-ACGGAGTGCCATCTGAAGAAG-3’  5’-GTAGCAGTGGAGCCAAGGTAG-3’  5’-GCTCCCTGTGTGTCTGATCTTG-3’  5’-GATATGCCCCAAGCCTATAAGC-3’  5’-CCTTCAATCTGCTGTGGTTCTTC-3’  5’-CTGTCTTCTTCAGAGTTGGAGTTG-3’  5’-CGAACGTGGAGGCGGTCTTAAC-3’  5’-TCTGTGATCTGCGGTTTGACTTGG-3’  5′-CTTGTGAAGATGACTCGGGATT-3′  5′-GATGTCAGTCTTGCTCCGTGA-3′  5′- GATGCCTACCGACGAAGACC-3′  5′-CGTGTCTGGCGTTGATTGATC-3′  5′-GTATCCACACAAGAGGCAGCATCC-3′  5′- ACAGCAGTTCAGCAGCAAGAAGG-3′  5′-TGCTGCCTTCCTTGGATGTG-3′  5′-TCGAACGTCTGCCCTATCAAC-3′  5′-ATGTTCTTGCTGAGTGTCTTCTTG-3′  5′-CTCTCTTGGCGATGGTTTCTTTC-3′  5′-CTGCTCAAGCGGTTGTCAATC-3′  5′-TATCTCATCATCTTCCTCCACCAG-3′  5′-GGATGAACAAGGTGCGGTGAGAC-3′  5′-CACTTCTGGGCAGCGAGACATC-3′  5′-TGACCCTCATTAGGAGTGGAC-3′  5′-GCTGCCTGCTTTCATTACC-3′  5′-GCAGTGGCTTTGAAGGGTTATAG-3′  5′-TGCTGTGCTTTGTATGAAGAGTG-3′  5′-GTTGTGTCAAGACTCCGTGGGTTC-3′  5′-CAGGGTGTGTAGGTTGGGCTATTG-3′  5′-TTCCTTCCTGATAGCAGCCATC-3′  5′-AGAGAAGCGTGAAGCGAGTG-3′  5′-CTGGGATTCACCTCAAGAACATC-3′  5′-CAGGGTCAAGGCAAGCCTC-3′  5′-CCAACCACCAGGCTACAGG-3′  5′-GCGTCACACTCAAGCTCTG-3′  5′-TTAAAAACCTGGATCGGAACCAA-3′  5′-GCATTAGCTTCAGATTTACGGGT-3′  5′-AAGCTGAGCGACGAGTACAAGA-3′  5′-GTCAGCTCCAGCACCTTGTG-3′  5′-GCTACCAAACTGGATATAATCAGGA-3′  5′-CCAGGTAGCTATGGTACTCCAGAA-3′  5′-GATTACTGCCCTGGCTCCTA-3′  5′-TCATCGTACTCCTGCTTGCT-3′  5′-GCTCATTTGCTCTTGGGTGGGCACTTCT-3′  5′-GAGCATGGCAGCAATCTTCAGAACAAGG-3′  5′-CCTGTTATGCCCACCACCAAAG-3′  5′-CCCTTGTTCTGAAGATTGCTGCC-3′  5′-TCTCTACCCTTCATCTTTCAAATGCC-3′  5′-ACATCCGTGACACACAGATTTGG-3′  5′-CTTTATTCAGCAAATGGAACATCCGTGACA-3′  5′-GTCCCGAGCGAGACGAG ACCAGGAGAAAC-3′  5′-CTCAGACAGCGAGGCACATCAG GTA-3′  5′- GCTTCAGATTTACGGGTCAACTTCACAT-3′ |

**Supplemental figure legends**

**Supplemental Fig. 1** Validation of lncRNA candidates by qRT-PCR. **a** qRT-PCR analysis of the top 12 upregulated lncRNAs in the mouse kidneys 24 hours after renal IR. **b** qRT-PCR analysis of the top 10 downregulated lncRNAs in the mouse kidneys 24 hours after renal IR. **c** Three lncRNAs were enriched in primary renal epithelial cells treated with hypoxia. Data represent mean ± SEM. n = 4.**P<0.01

**Supplemental Fig. 2** Nucleotide sequence of the full-length lncRNA-IRAR gene.

**Supplemental Fig. 3** Flow cytometric analysis on infiltration of nertrophils and macrophages in kidneys after ischemia reperfusion. Data represent mean ± SEM. n = 6.**P<0.01

**Supplemental Fig. 4** IRAR increases proinflammatory cytokine production and apoptosis in HK-2 cells. **(A)** qRT-PCR analysis of *IRAR* in HK-2 cells transfected with *IRAR* overexpressing lentivirus (LncRNA OE) or Negative lentivirus (LncRNA NC). Data represent mean ± SEM. n = 4.**P<0.01. **(B-D)** qRT-PCR analysis of CXCL1 (B), CXCL2 (C), CCL2 (D) in hypoxia-treated HK-2 cells transfected with *IRAR* overexpressing lentivirus or Negative lentivirus. **(E)** qRT-PCR analysis of IL-6 expression. **(F)** Cell apoptosis assay by flow cytometry for hypoxia-treated HK-2 cells transfected with *IRAR* overexpressing lentivirus or Negative lentivirus. Data represent mean ± SEM. n = 4. **P<0.01.

**Supplemental Fig. 5** Knockdown of *IRAR* suppresses chemokines expression and apoptosis in hypoxia-treated HK-2 cells. **(A)** qRT-PCR analysis of *IRAR* in hypoxia-treated HK-2 cells transfected with GapmeR *IRAR* or Control. **(B-D)** qRT-PCR analysis of CXCL1 (B), CXCL2 (C), CCL2 (D) in hypoxia-treated HK-2 cells transfected with GapmeR *IRAR* or Control. **(E)** qRT-PCR analysis of IL-6 expression. **(F)** Cell apoptosis assay by flow cytometry for hypoxia-treated HK-2 cells transfected with GapmeR *IRAR* or Control. Data represent mean ± SEM. n = 4. **P<0.01.

**Supplemental Fig. 6** Immunohistochemical staining for CCL2 in kidneys 24 hours after renal IR. T indicates renal tubules, G indicates glomeruli. Black arrows indicate CCL2-expressed tubular epithelial cells.

**Supplemental Fig. 7** Prediction of the transcription factor and binding sites on lncRNA-IRAR promotor. TSS, transcription starting site.
