# Supplementary figures and images for: LncRNA IRAR regulates chemokines production in tubular epithelial cells thus promoting kidney ischemia-reperfusion injury
Source: Cell Death Dis. 2022 Jun 22;13(6):562. doi: 10.1038/s41419-022-05018-x (PMC9217935; doi:10.1038/s41419-022-05018-x)

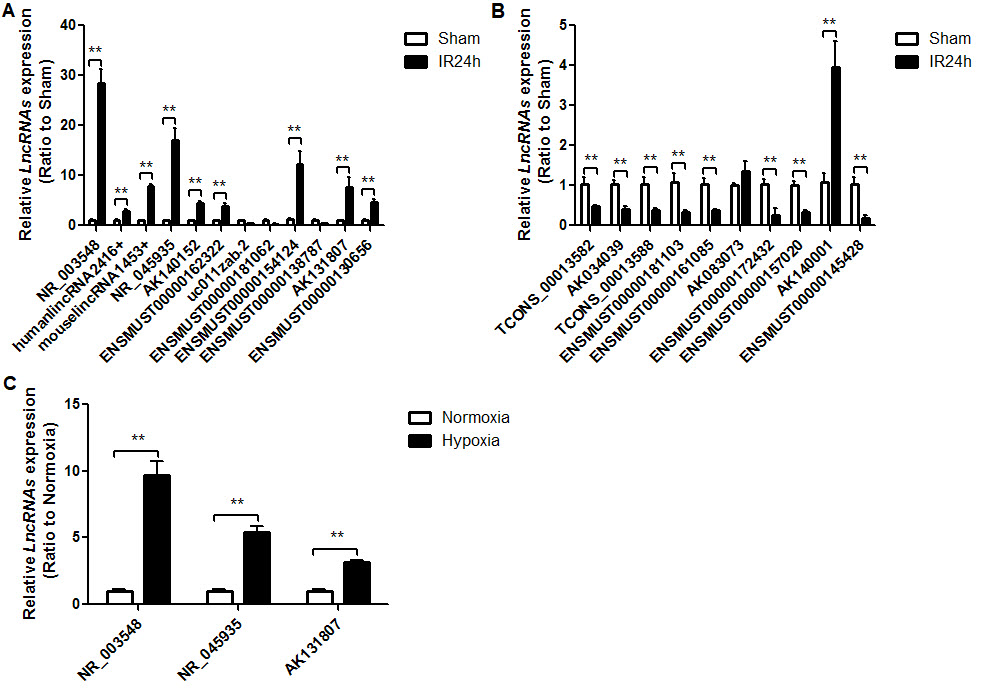

Supplement: Supplementary file 2 — Supplemental Fig. 1 [file 41419_2022_5018_MOESM2_ESM.jpg]

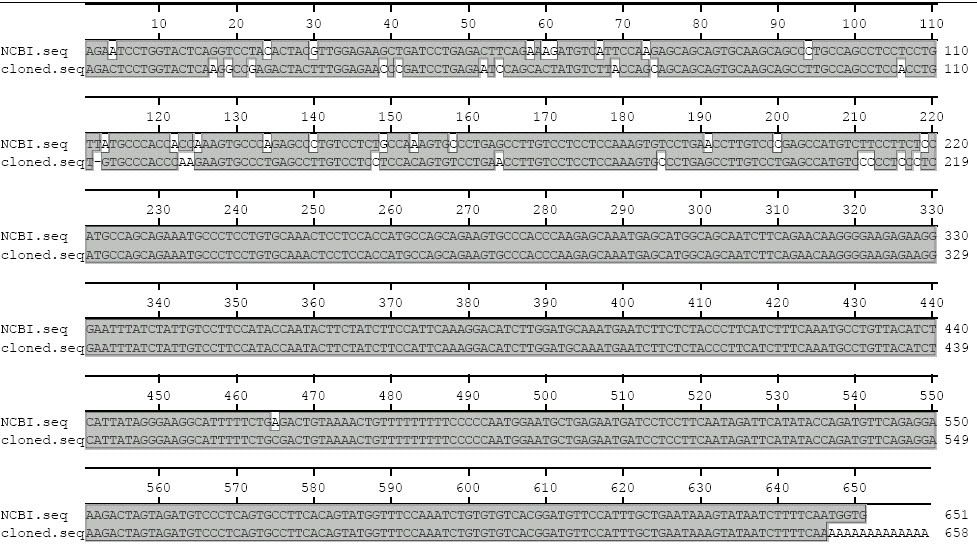

Supplement: Supplementary file 3 — Supplemental Fig. 2 [file 41419_2022_5018_MOESM3_ESM.jpg]

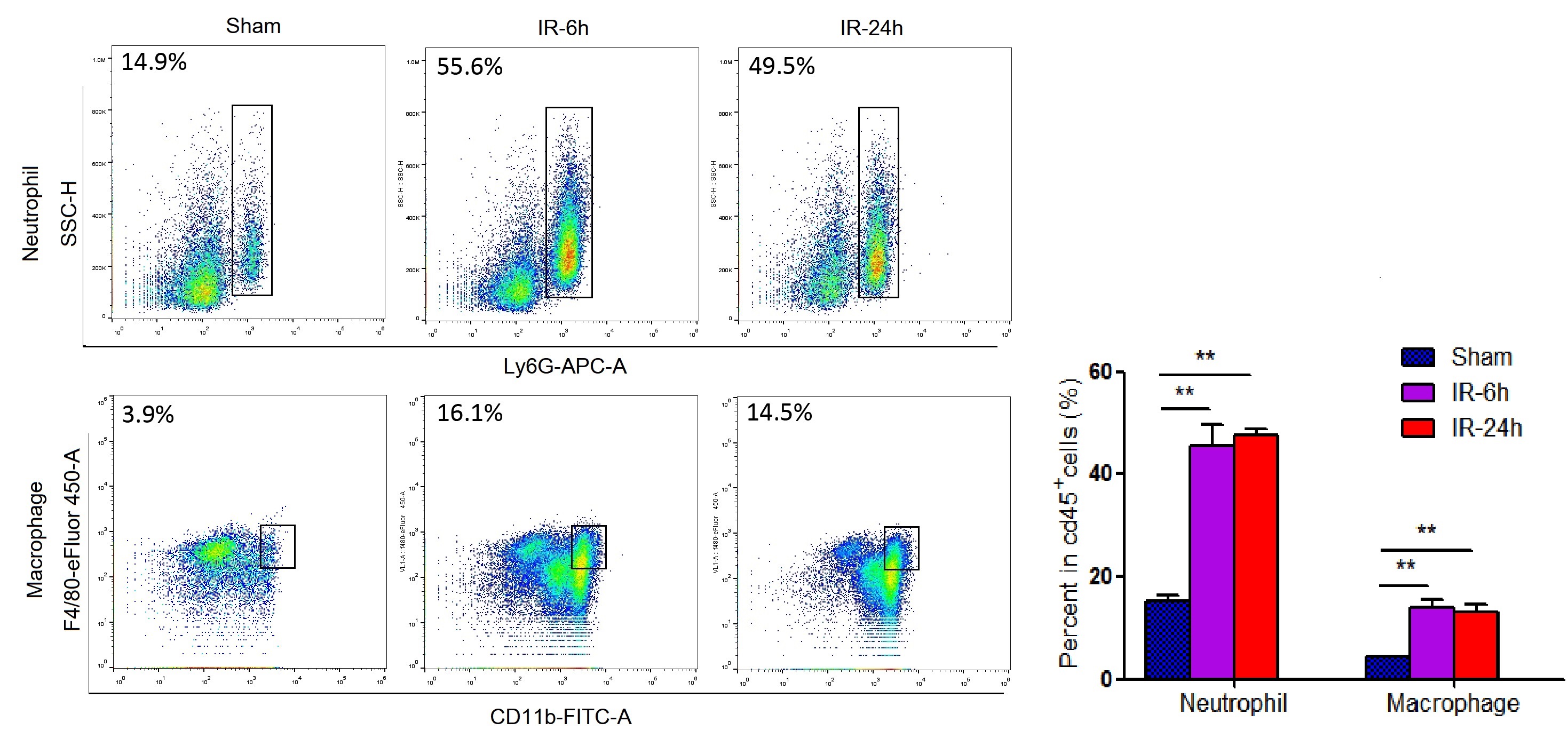

Supplement: Supplementary file 4 — Supplemental Fig. 3 [file 41419_2022_5018_MOESM4_ESM.jpg]

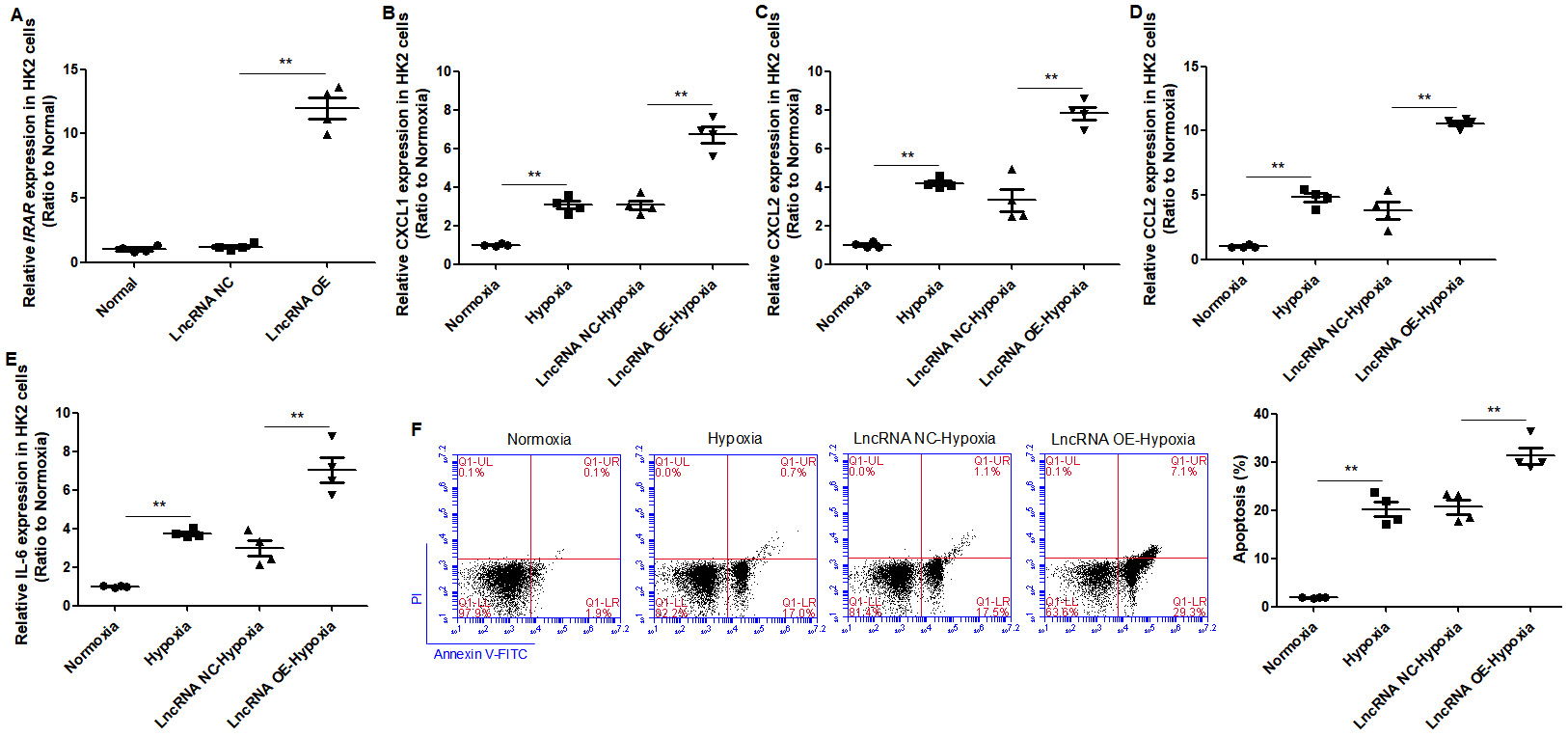

Supplement: Supplementary file 5 — Supplemental Fig. 4 [file 41419_2022_5018_MOESM5_ESM.jpg]

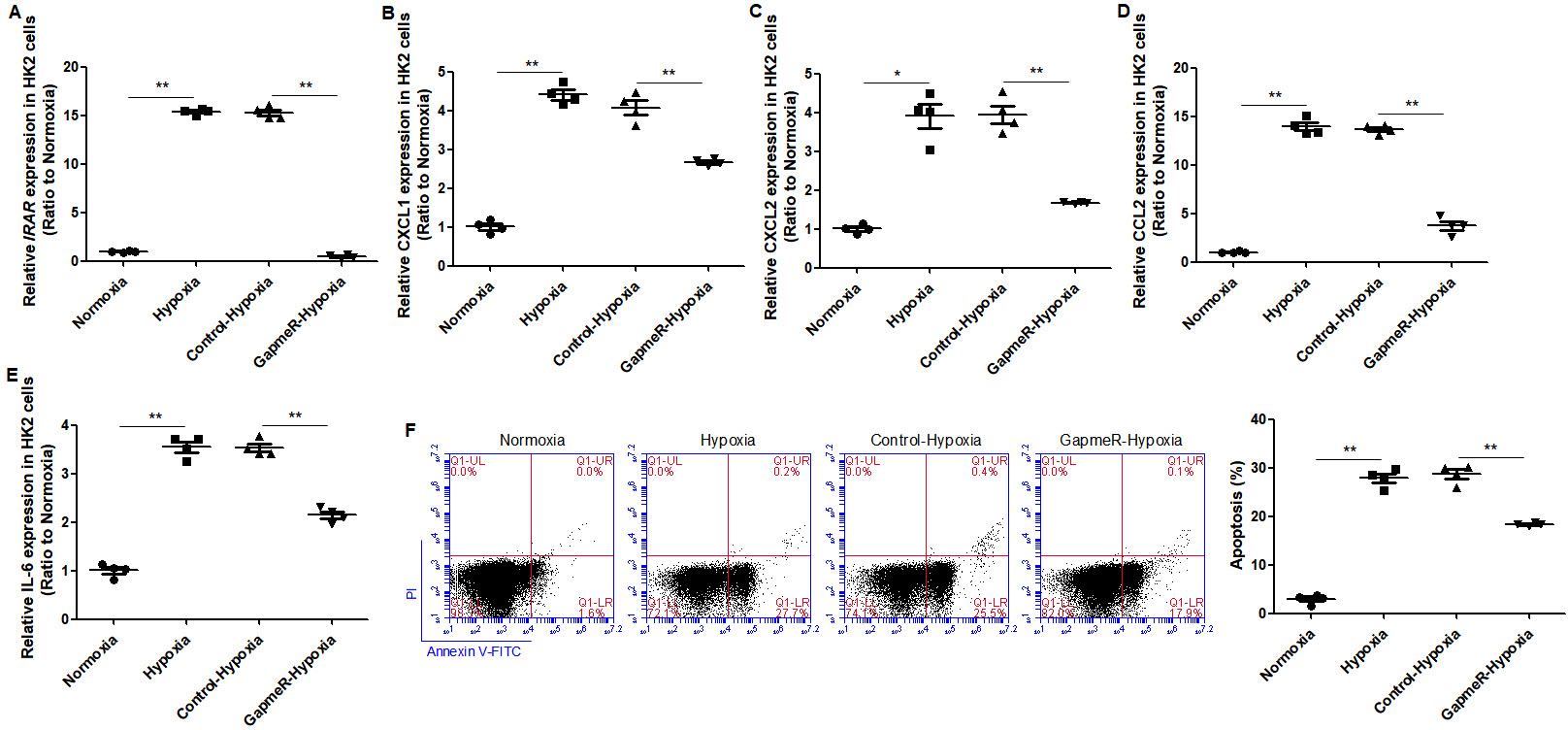

Supplement: Supplementary file 6 — Supplemental Fig. 5 [file 41419_2022_5018_MOESM6_ESM.jpg]

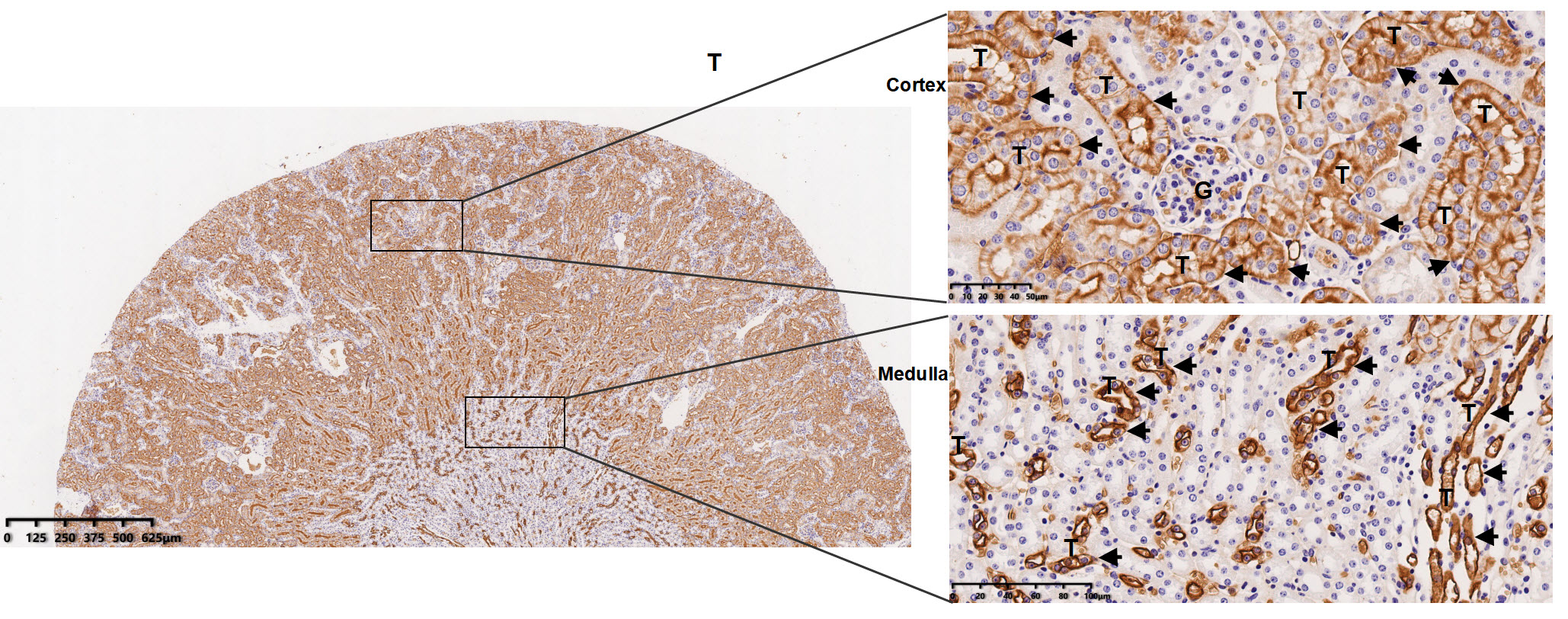

Supplement: Supplementary file 7 — Supplemental Fig. 6 [file 41419_2022_5018_MOESM7_ESM.jpg]

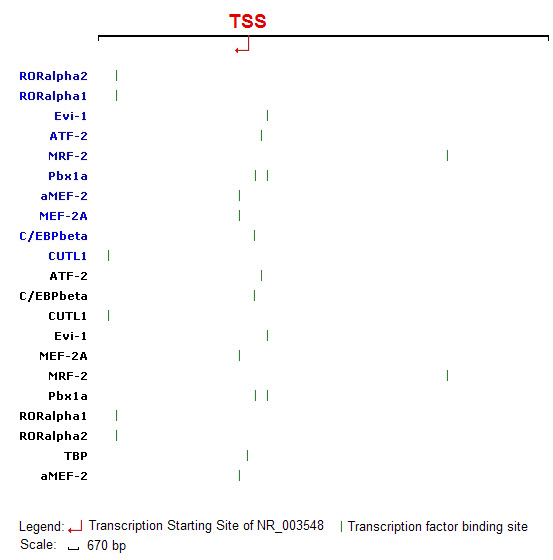

Supplement: Supplementary file 8 — Supplemental Fig. 7 [file 41419_2022_5018_MOESM8_ESM.jpg]
