# Supplementary material for: LncRNA IRAR regulates chemokines production in tubular epithelial cells thus promoting kidney ischemia-reperfusion injury
Source: Cell Death Dis. 2022 Jun 22;13(6):562. doi: 10.1038/s41419-022-05018-x (PMC9217935; doi:10.1038/s41419-022-05018-x)
Supplement: Supplementary file 9 — full and uncropped western blots [file 41419_2022_5018_MOESM9_ESM.pdf]

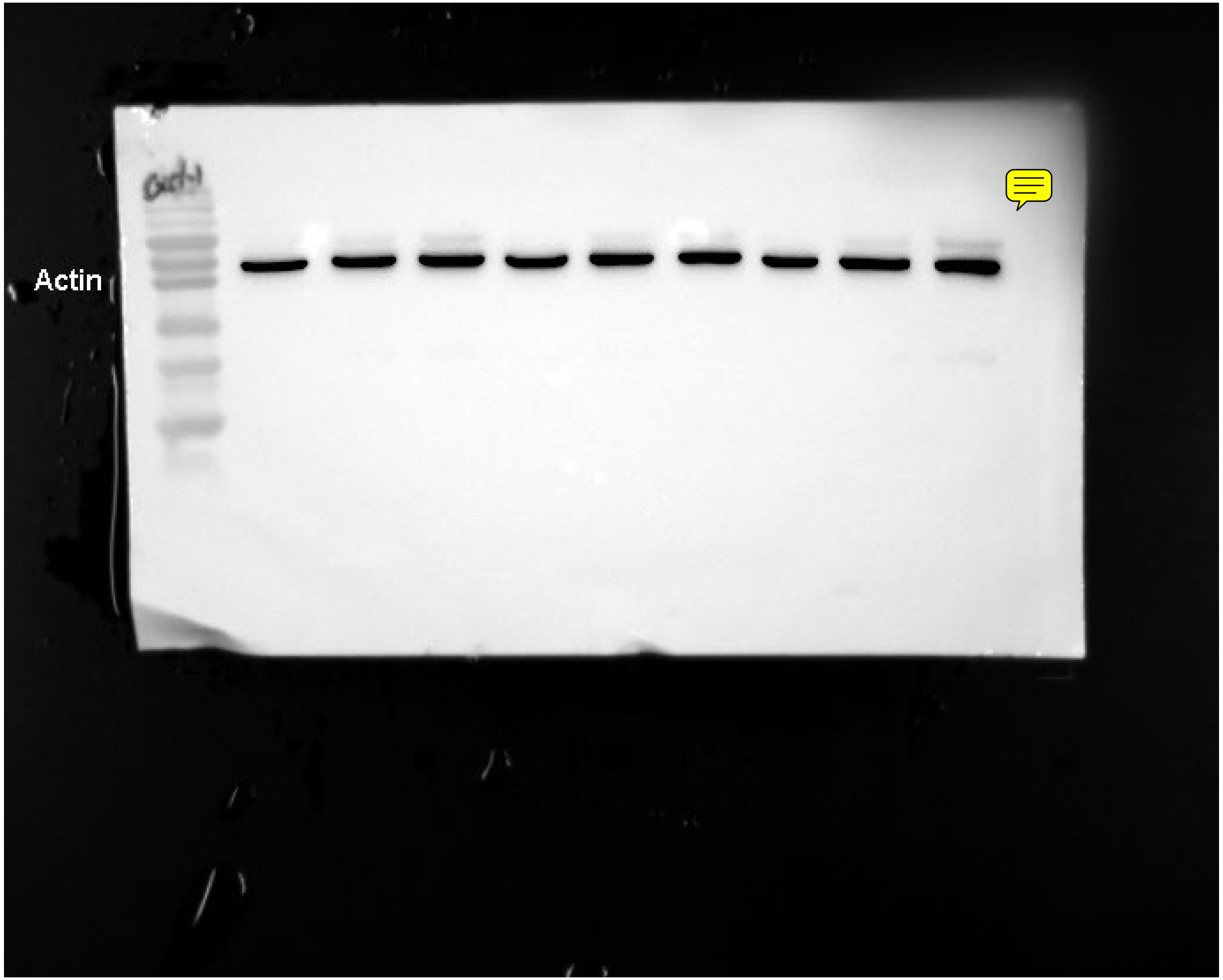
Actin

CCL2

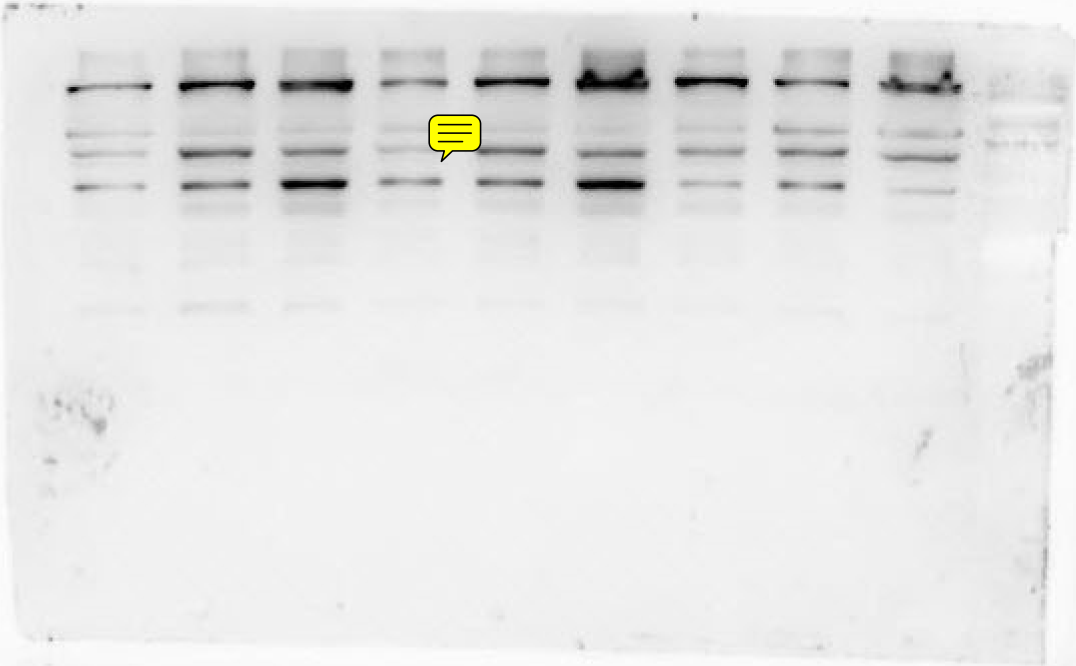

CXCL1

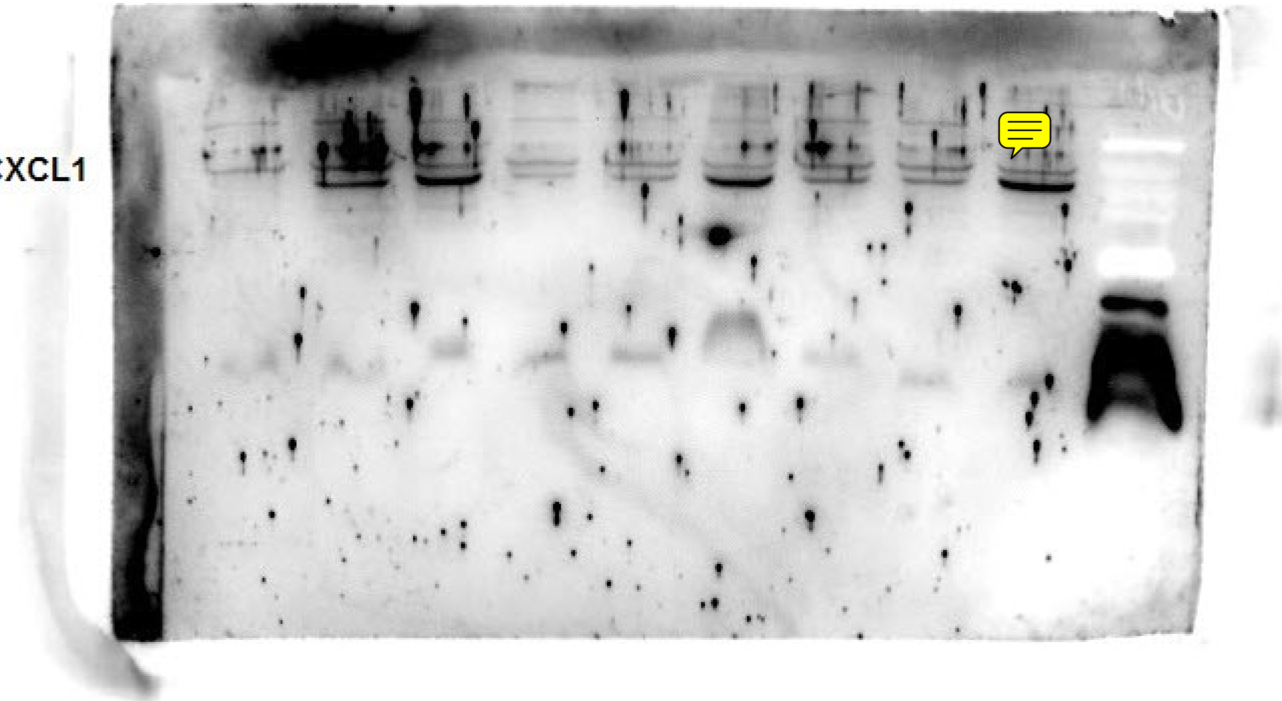

CXCL2

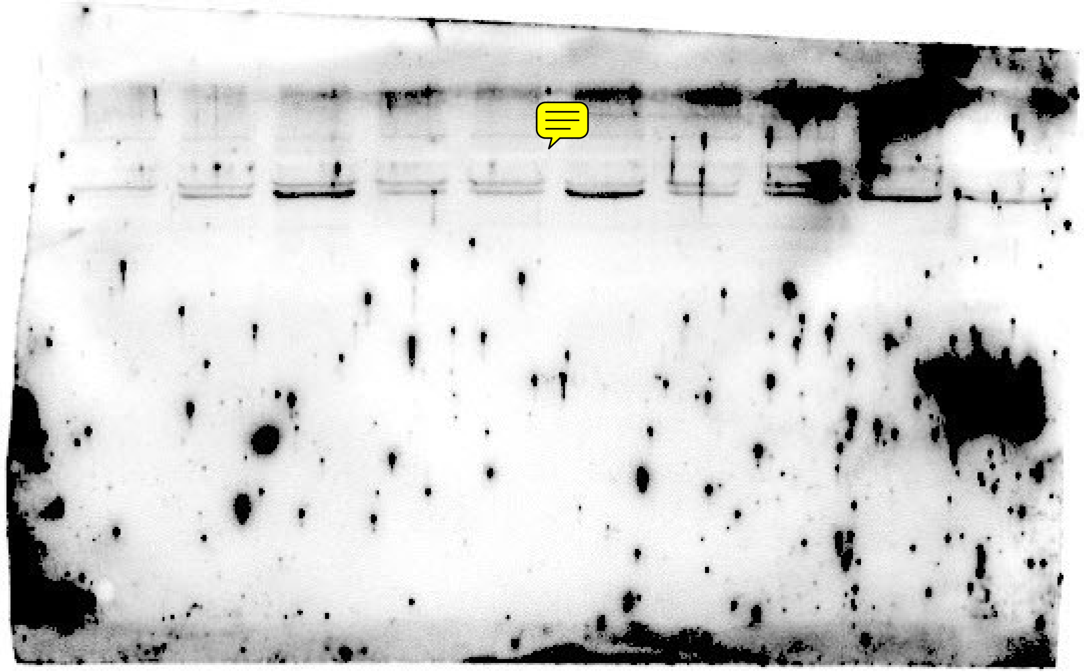

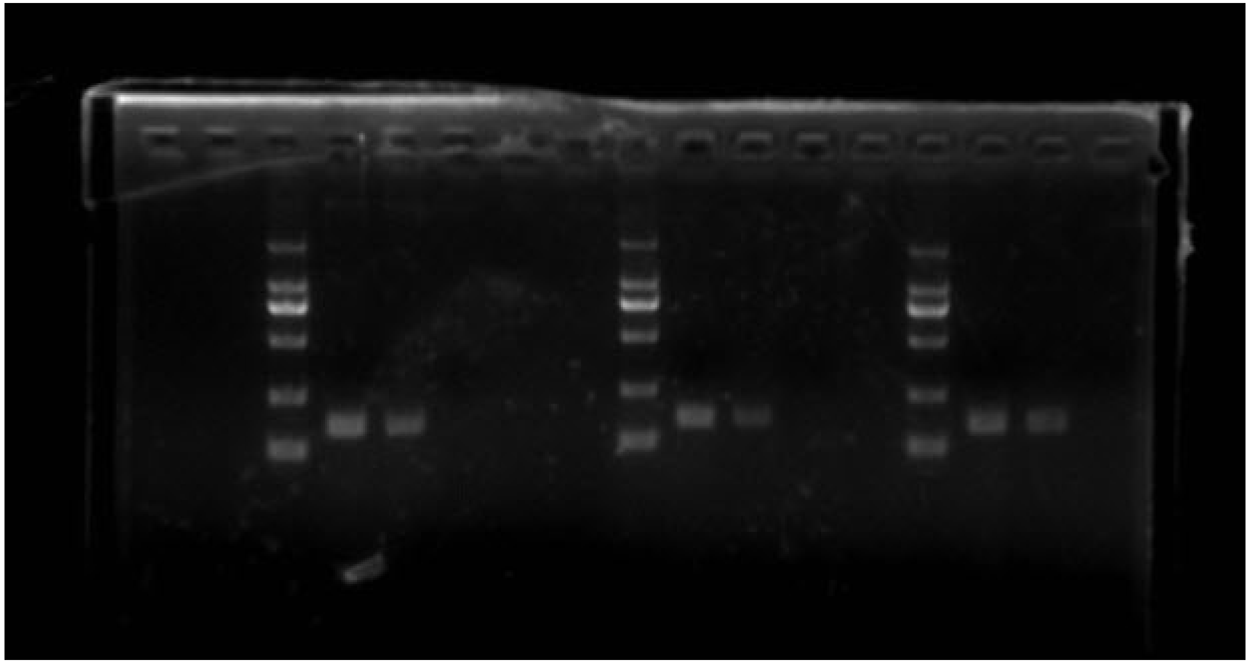

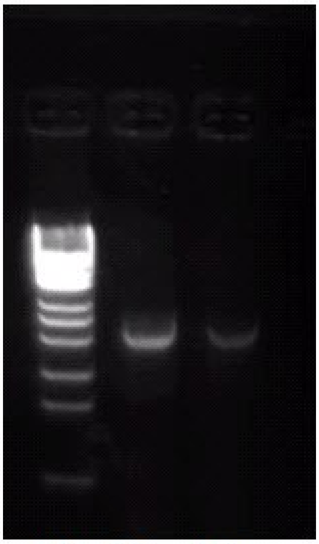

M L1 L2

M： DNA Marker

L1： biotin labeled LncRNA（~600bp）

L2： biotin labeled antisense LncRNA（~600bp)

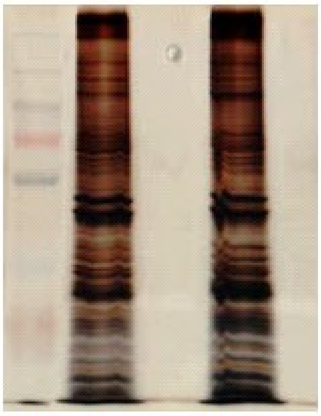

M L1 L2
Silver staining

M: DNA marker
L1: antisense RNA pull down
L2: RNA pull down

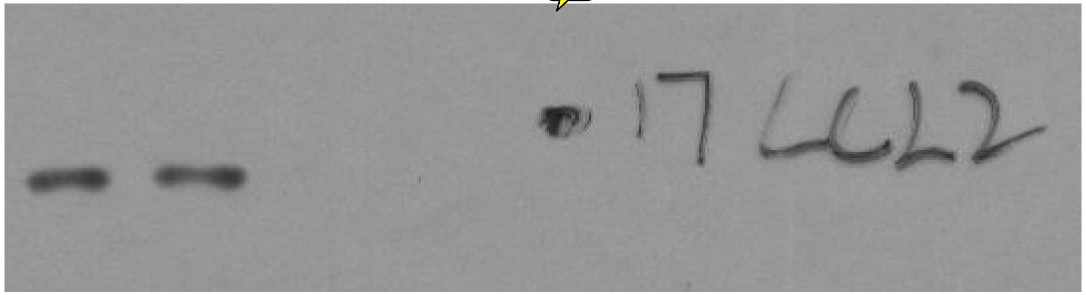
17 LCL2

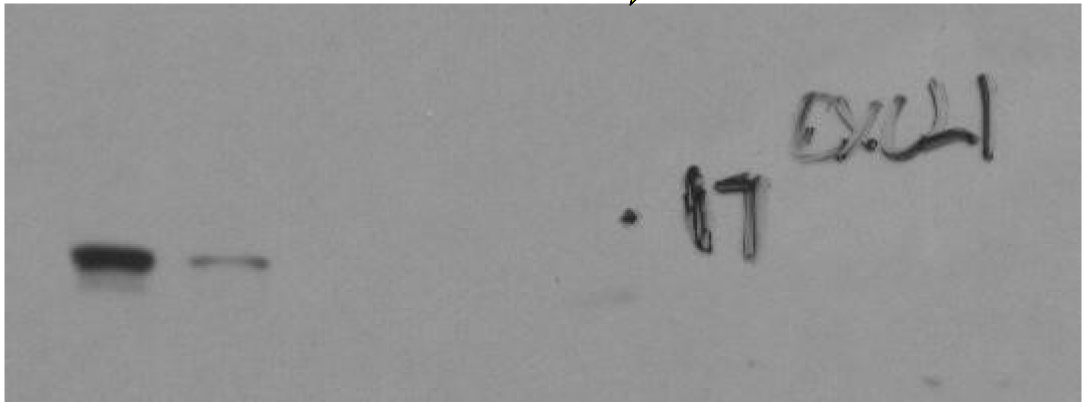
17

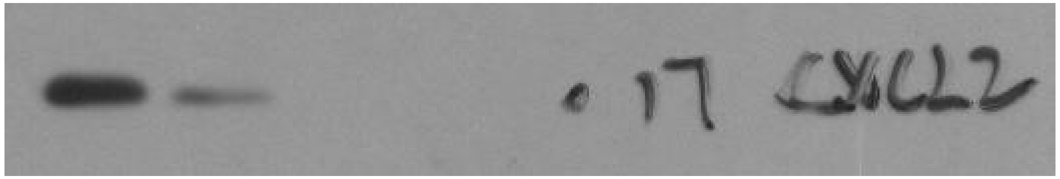
17

CCL2

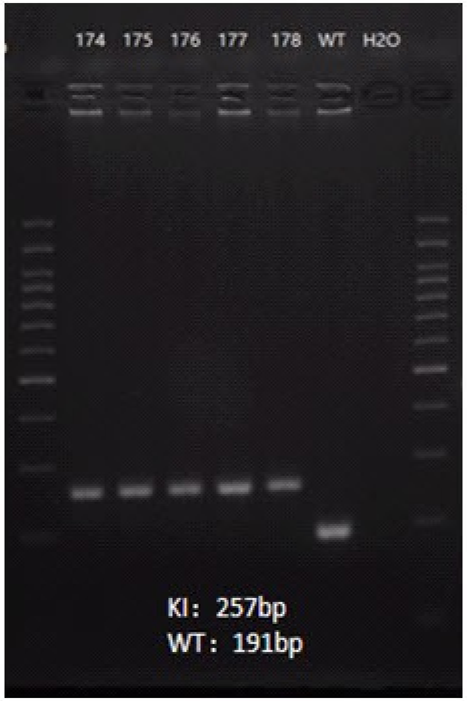

Cdh16 cre

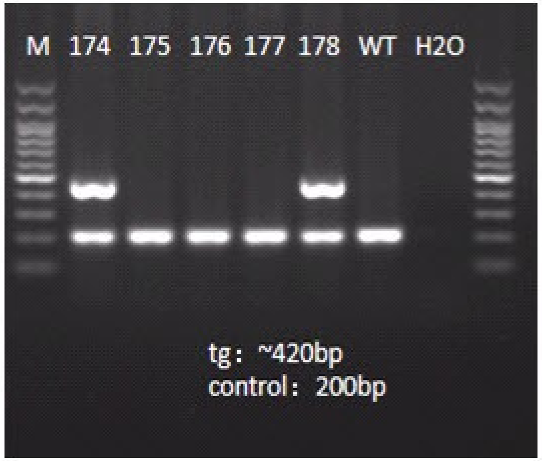

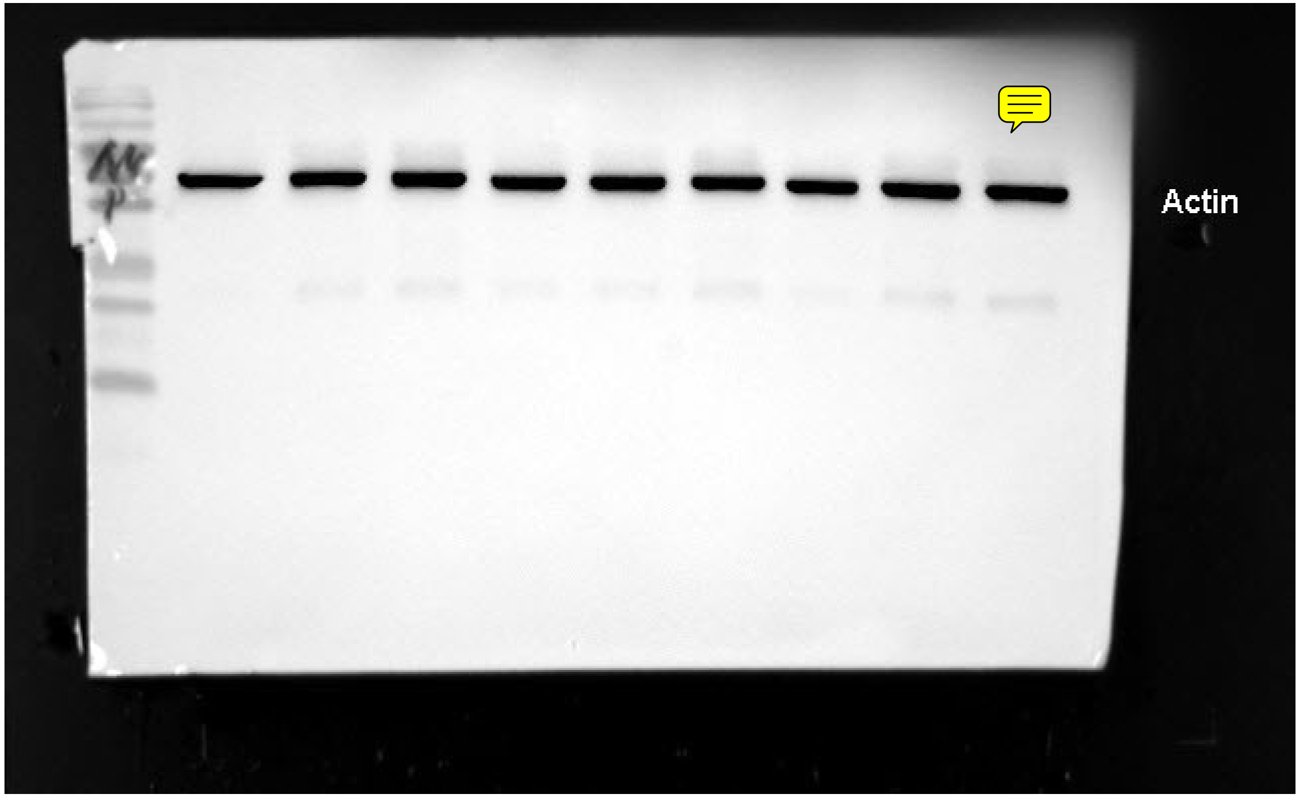
Actin

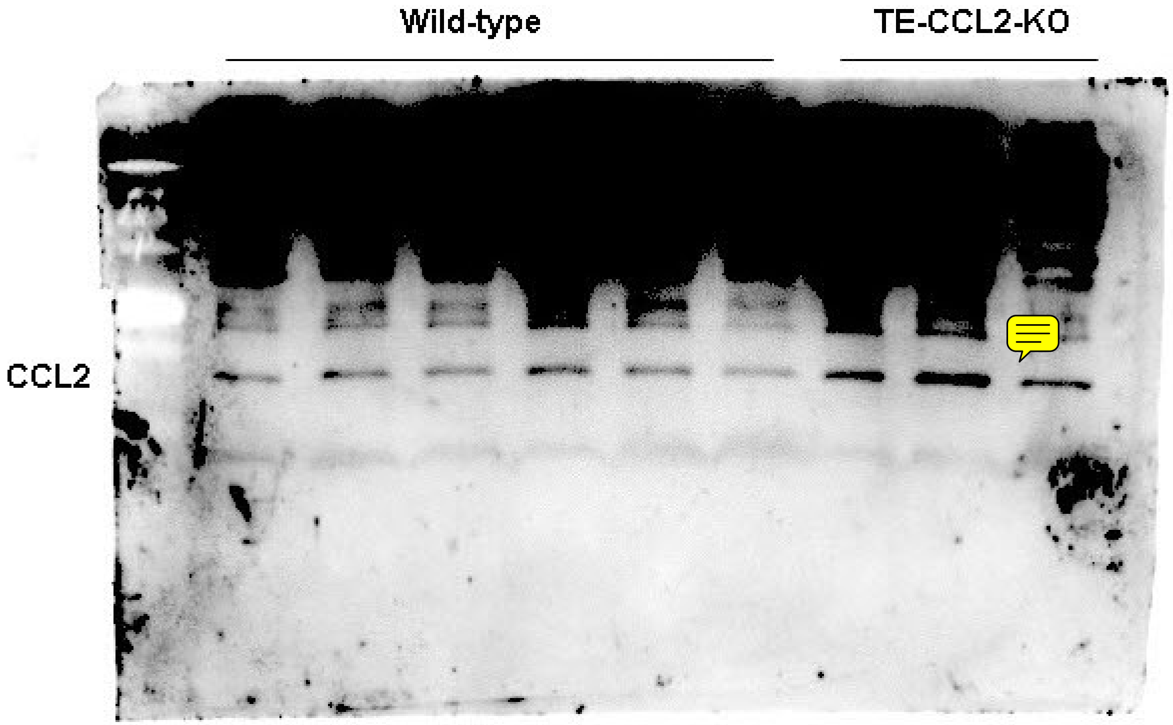
Wild-type
TE-CCL2-KO
CCL2

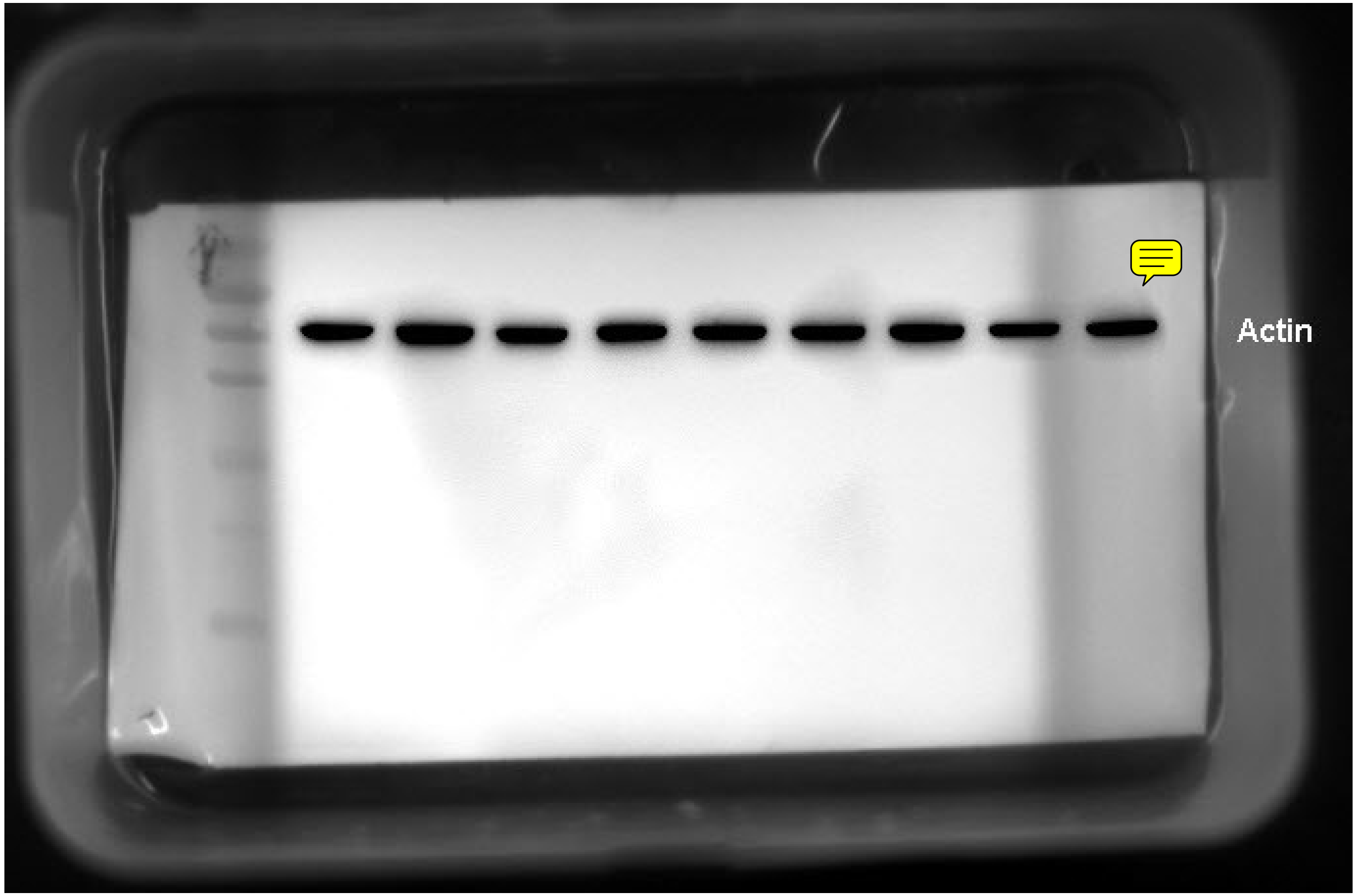
Actin

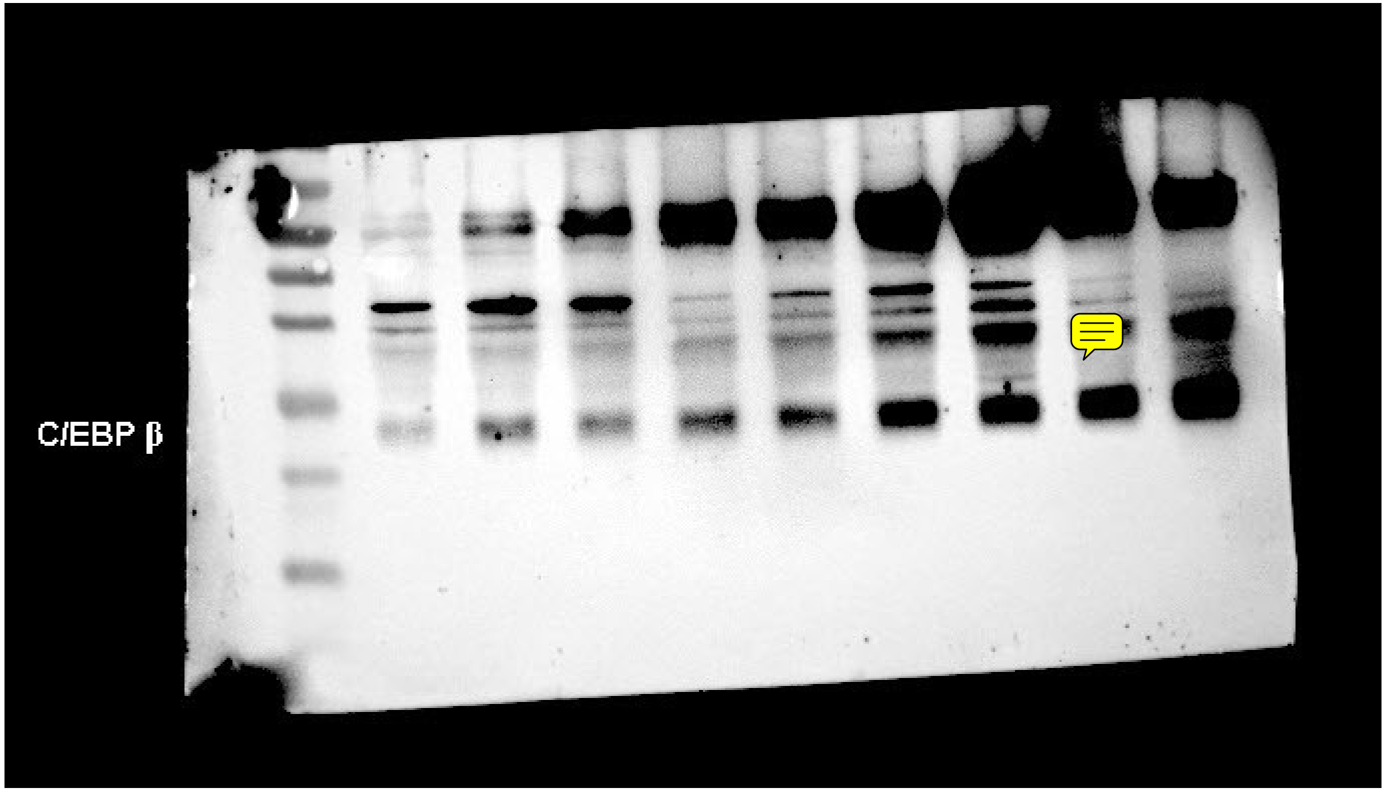
C/EBP β

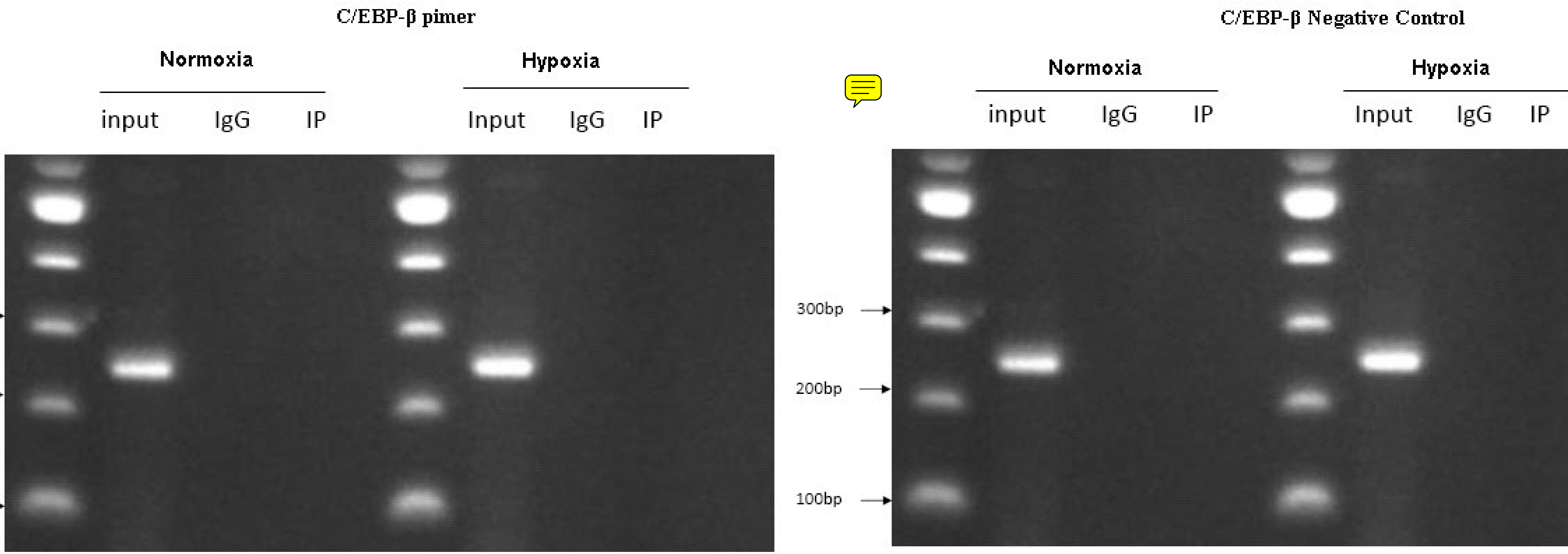

C/EBP-β pimer
Normoxia
Hypoxia
input
IgG
IP
Input
IgG
IP
C/EBP-β Negative Control
Normoxia
Hypoxia
input
IgG
IP
Input
IgG
IP
300bp
200bp
100bp
